# Community and facility-level barriers to achieving UHC in Kono District, Sierra Leone and Maryland County, Liberia

**DOI:** 10.1371/journal.pgph.0002045

**Published:** 2023-06-26

**Authors:** Julia Higgins, Jean Gregory JEROME, Foday Boima, Emily Dally, Luke Krangar, Emma Jean Boley, Sterman Toussaint, Yusupha Dibba, Chiyembekezo Kachimanga, Michael Mhango, Vivian Chung, Samuel Watson, one behalf of the Impact Consortium

**Affiliations:** 1 Partners In Health, Boston, Massachusetts, United States of America; 2 Partners In Health—Sierra Leone, Kono District, Sierra Leone; 3 Partners In Health—Liberia, Harper, Liberia; 4 Partners In Health—United States, Boston, Massachusetts, United States of America; 5 Partners In Health—Malawi, Neno, Malawi; 6 Institute of Applied Health Research, University of Birmingham, Birmingham, England; Indian Council of Medical Research, INDIA

## Abstract

Universal Health Coverage (UHC) is achieved when individuals and communities receive the health services they need without suffering financial hardship. However, many countries face barriers to building health systems that enable the availability of affordable, accessible care. The goal of this study was to present a model of local monitoring of barriers and to provide a roadmap for designing interventions that improve access to and use of healthcare delivery systems. We conducted household, individual, and health facility surveys in seven catchment areas in Sierra Leone and Liberia between December 2019 and March 2020. A two-stage cluster sampling method was used to sample households and individuals, and all health facilities were included. We divide access barriers into demand (patient-side care seeking behavior), supply (availability of facilities and services), and their intersection (affordability, spending, and use rates). Among the 2,576 respondents within our 1,051 surveyed households, the propensity to seek care when ill was reported at 90% in Sierra Leone (n = 1,283) and 70% in Liberia (n = 806). We estimated that 31% of households spent greater than 10% of their total expenditure on healthcare in a month, and that 14.5% of households spent greater than 25%. Overall, the general service readiness index mean score for all health centers was around 70%. The greatest hindrance to service readiness was the availability of essential medicines, with facilities reporting an average score of 32% in Sierra Leone and 63% in Liberia. Our evidence suggests that the cost of care is both a barrier to care-seeking and a persisting problem among care-seeking patients. Lack of service availability (essential equipment and medicines), poses a risk to high-quality care. The research team recommends deploying interventions (visit cost subsidies, supply chain improvements) targeted at resolving these issues in order to advance the goal of achieving UHC.

## Introduction

Universal health coverage (UHC) is defined as individuals and communities receiving the health services they need without suffering financial hardship [1]. Achieving UHC is one of, and is central to, the health-related targets of the Sustainable Development Goals (SDG), codified as target 3.8. Two indicators were developed to monitor progress towards this goal: SDG 3.8.1, which looks at the coverage of essential services, and SDG 3.8.2, which looks at the proportion of households with large health expenditures as related to total household budget [2–4].

The use of available health services results from the confluence of both demand (patient) and supply (health service) side factors [5]. Patients must recognize symptoms and choose to seek care with appropriate providers, while clinics and hospitals must provide high-quality services at affordable rates in order for patients to receive care that their conditions require in a timely manner. There is a multitude of possible barriers on both demand and supply sides that can prevent patients from receiving timely and affordable care, as such, no UHC program can be “one size fits all.” Monitoring and surveillance are therefore required to identify UHC barriers and appropriately design interventions to improve access to and use of health care delivery systems [6].

There are several widely used indicators of UHC, such as use rates of different services and their relationship with household wealth or income and catastrophic health expenditure. However, such statistics are generally reported at the national level and provide little insight into within-country variation or reasons for levels of performance at local levels [7–9]. For example, low vaccination rates in one area may be due to patient hesitancy or lack of knowledge, or be due to a poor supply chain, lack of cold storage at facilities, or a lack of staff to deliver them. Thus, more in-depth studies at a local level are required to complement aggregate statistics, particularly because there may be significant variation between areas. For example, recent evidence suggests that, in many cases, there may be more variability of access to and use of care within local areas than between them [10]. As another example, national estimates of catastrophic health expenditure may underestimate financial hardship in poor households and overestimate it in rich households, and low rates of catastrophic health expenditure may just reflect poor service coverage [11, 12].

This paper presents an exemplar of local monitoring and tracking of community and facility-level barriers to achieving UHC. We examined care-seeking and access in communities and facilities where care delivery is supported by the non-profit organization Partners In Health (PIH): Kono District, Sierra Leone, and Maryland County, Liberia. Following recent crises, including lengthy civil wars and the Ebola epidemic, the WHO’s UHC Service Coverage Index [13], which tracks national-level progress towards SDG 3.8.1, reports that as of 2019, only 39% of the country has achieved UHC within Sierra Leone, along with 42% in Liberia. PIH aims to strengthen the delivery of services and expand access to health care in partnership with the Ministry of Health and Sanitation (MoHS) of Sierra Leone and the Ministry of Health (MOH) of Liberia through investments in the fundamental elements of health system strengthening: staff, ‘stuff’, space, systems, and social support. PIH provides additional health care workers for direct patient care, clinical and community health worker training, supply chain support, innovative clinical interventions bringing modern technology/science closer to those who need it the most, and financial support for food, housing, and transportation to support patients and their families to foster access to care and improve health outcomes. PIH’s work in West Africa began as targeted Ebola response work in 2014, followed by engagement in communities in Maryland County and Kono District in 2015. PIH Sierra Leone launched a Primary Health Center (PHC) expansion project in 2019, the rollout of which aligns with the timeline of this study. We conducted the research reported here to inform where and how to intervene to improve UHC in a way that reflects the needs and demands of the population along with the limitations and barriers of the existing health service.

## Methods

### Ethics statement

In Sierra Leone, the Sierra Leone Ethics and Scientific Review Committee (SLESRC) granted ethical approval for this study (study title: Evaluation of Primary Care Health System Strengthening Intervention in Kono District, Sierra Leone). In Liberia, the University of Liberia-Pacific Institute for Research & Evaluation Institutional Review Board (UL-PIRE IRB) granted ethical approval for this study (protocol #: 19-10-183).

### Setting/ study population

**Fig 1:**
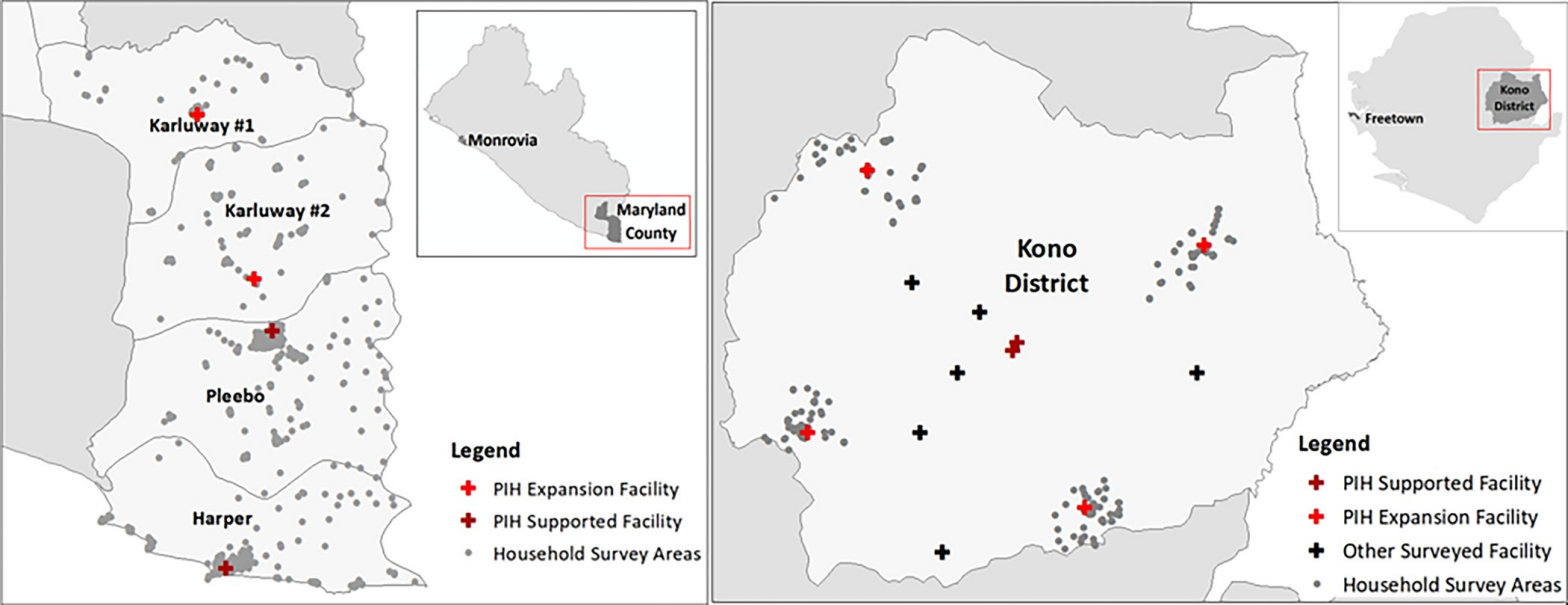
Household survey areas and their geographic relation to PIH-supported facilities in Maryland County and Kono District (ArcGIS online, Sierra Leone and Liberia).

UHC outcomes result from the intersection of patient and population (demand-side) choices and behaviors, and facility and system (supply-side) characteristics and services, so we designed surveys for both aspects of the system to contextualize and interpret commonly used statistics including care-seeking, use rates, and satisfaction. We conducted household, individual, and health facility surveys in four catchment areas covered by 15 health facilities, including 11 facilities in Kono District, Sierra Leone, and four areas covered by four facilities in Maryland County, Liberia where care delivery is supported by PIH. The household and individual surveys required participants to recall information about their prior health seeking behaviors. Fig 1 below displays household survey areas and their geographic relation to PIH-supported facilities in Maryland County and Kono District.

The research team chose the surveyed facilities purposefully with the guidance from the regional health officers in both countries. The 15 sites include 14 public primary health facilities and 1 public/private facility (Wellbody Clinic) that provide a range of services such as basic antenatal and obstetric care, tuberculosis, malaria, and HIV services. They all serve relatively large catchment areas and support smaller primary health units (PHUs) within their catchment areas. All sites were rural and within reasonable travel distance of the referral hospital in the regions of the two countries. Transit options in both Kono District and Maryland County are limited and often act as a barrier to care in our research settings. In order to better understand this barrier, we collected information on how frequently individuals were able to utilize private vehicles, public transportation, taxis, ambulances/emergency vehicles, bicycles, motorcycles, walking, and other sources of transit when seeking healthcare.

The team implemented the household and individual surveys from November 2019 –March 2020 in Sierra Leone and March 2020 and April—May 2021 in Liberia. The health facility assessments were completed from November 2019—March 2020 in Sierra Leone and September 2021 in Liberia. The field teams implemented the household and individual surveys concurrently and conducted the health facility assessment separately. The team electronically programmed both instruments in CommCare and administered the tools using Android tablets. The research team strategically chose the survey tools to ensure their focus areas complemented one another, given that one set of tools collects household-level data and the other tool aggregates facility-level information. Though this study has demonstrated the need for health sensitization and education programming to encourage health-seeking behavior, to the best of our field teams’ knowledge, there were no information, education, or communication (IEC) activities occurring during the period of the study that may have affected respondent behavior.

### Study design and procedures

#### Household and individual surveys

Local teams implemented community-based household and individual surveys in all catchment areas between November 2019 and September 2021.

The sampling procedure aimed to sample and survey 500 households across the four catchment areas (Sewafe, Kombayende, Gandorhun, and Kayima) in Sierra Leone and 500 in the three catchment areas (Harper, Pleebo, and Karluway 2) in Liberia. In the absence of a reliable sampling frame, we used a two-stage cluster sampling method. We used the most recently available satellite imagery for the area to define geographic clusters of structures [14]. The clusters were randomly sampled to ensure that they were well spread out across the district. The number of sampled clusters per catchment area was proportional to population size. For the second stage, all households in sampled clusters were then enumerated and included in the study (sometimes referred to as “compact segment sampling” when used by UNICEF MICS and other surveys) [15–18]. Up to three attempts were made to survey each household.

A household roster containing demographic information about each household member was completed by the head of each household–in our research context, defined as the individual who holds the most information about the residents who live in the structure that is being surveyed. From the completed household roster, a random adult man (aged 15–49), adult woman (15–49), and child under five (0–59 months) was selected, if any were resident, to complete the individual surveys. Adult caretakers completed surveys on behalf of the selected child. All adolescent and adult participants provided consent, and the research team sought consent from the primary caregiver for under five participants. Participants were considered ineligible if at the time of the survey they were imprisoned, homeless or visiting from outside the survey catchments areas for less than 30 days, or if they are otherwise unable to give informed consent. Polygamous and multi-household structures were distinguished by separate eating and cooking facilities. The household interview was completed preferentially by the head of the household.

All questionnaires were adapted from existing World Health Organization (WHO) and Demographic and Health Surveys (DHS) tools [19]. Information on barriers to care were collected utilizing a series of multi-select questions embedded in the household survey; if an individual did not seek care when ill, they provided a reason for why they were deterred from seeking treatment, which was then categorized as a barrier to care seeking by the research team. All study staff engaged in data collection received a comprehensive five-day training, including one day of field-testing. Field teams trained 10 enumerators and three supervisors on the questionnaire content in Sierra Leone, along with 28 enumerators in Liberia. In terms of enumerator recruitment and quality assurance, site teams targeted hiring individuals with experience collecting DHS and local census data. In Sierra Leone, enumerators participated in a 6-day training exercise and conducted extensive pilot testing of the questionnaires in villages adjacent to those sampled for the survey. In Liberia, enumerators were administered pre and post tests to assess the impact of their training on their survey-related knowledge and competencies (individuals who scored 80% or higher were cleared to participate in fieldwork). All recruited enumerators across sites were fluent in relevant local languages, particularly Grebo (Liberia) and Krio (Sierra Leone).

The household survey captured data on demographic and socioeconomic status of household residents, as well as the Demographic and Health Survey (DHS) wealth index and household expenditure. Individual surveys were designed to capture need for healthcare, access to and use of healthcare facilities, as well as healthcare knowledge and health status. To consider access, we asked respondents if they had experienced any acute illness symptoms in the previous month and their severity, and then whether any care was sought for these symptoms. For respondents not reporting care-seeking we asked about the last time they had sought care. We also asked about routine care for chronic conditions, disease-specific knowledge and behavior, child vaccinations, and self-reported health.

#### Health facility assessment

The World Health Organization Service Availability and Readiness Assessment (SARA) survey tool was used to assess the availability of basic services as well as facility readiness to provide those services [20]. All SARA survey components were utilized, including service availability, general service readiness, and specific service readiness. The survey covered the following service areas: general facility status, basic equipment, human resource for health, drugs and supplies, standard infection prevention practices, diagnostic services and some specialized services including their location and functional status and components of support systems (e.g., logistics, maintenance, and management).

Upon arrival at each facility, informed consent was obtained, GPS coordinates were collected, and the survey was administered to the most knowledgeable person at the facility for each section of the questionnaire. To prepare for data collection, six members of the field team attended three days of survey training conducted by a lead member of the Sierra Leone and Liberia implementation teams.

#### Outcomes and analysis

Following recent large-scale studies of UHC, we divided our indicators into several categories: care seeking and service utilization, barriers to care, healthcare spending, and facility readiness and availability. Survey weights were used in all statistical summaries to reflect the sampling process. The survey weights were defined as the inverse probability of being included in the sample: we replicated the sampling process at the cluster-level 10,000 times to estimate the probability each cluster would be included in the sample, which was not equal as the sampling method favored samples with geographically dispersed clusters; at the individual-level the probability of being in the sample was equal to the inverse of the number of eligible individuals for each survey. The "second stage" in our two-stage sampling process consisted of enumerating and "sampling" all residents in the segment/cluster we identified for inclusion in fieldwork.

#### Care seeking and service utilization

For care-seeking, we estimated the proportion of patients at the catchment area level with self-reported severe acute illness who: i) sought care; ii) sought care at a facility; and iii) sought care with a trained health provider at a facility. This information was collected as part of the household survey, in which we asked respondents to recall their previous experiences of falling ill and seeking care when sick. We utilize this ‘cascade’ of health seeking behavior to contextualize how patients seek and receive treatment: those categorized as ‘seeking treatment’ received care from a provider in some capacity, even if that capacity was outside the formal healthcare system and was provided by an untrained practitioner or at a mobile clinic or outreach event. Respondents categorized as those ‘receiving care at a facility’ explicitly visited a health facility/center for care as opposed to being seen by an informal provider or at a mobile clinic/outreach event. Lastly, those categorized as ‘receiving care from a trained healthcare provider’ saw a doctor, nurse, community health officer, or dentist once they arrived at a facility (as opposed to visiting a facility for care and being treated by an untrained provider or not being seen at all). ‘Acute illness’ indicates that a respondent experienced any of the following minor, non-chronic illnesses within the month preceding the survey date: diarrhea, fever, difficulty breathing, injury, pain, skin problems, anxiety/depression/difficulty sleeping, nausea/dizziness, appetite problems, fatigue, or ‘other’. Episodes of acute illness were classified as ‘severe’ if the respondent identified their illness as affecting their daily life either ‘extremely’ or ‘a lot’ on a Likert scale. The survey tools elicited information about acute illness from the three individual respondents randomly selected from the household roster. We also estimated annual use rates and proportions across several healthcare areas: annual outpatient consultation rates, annual inpatient discharge rates, proportion of pregnant women receiving four or more antenatal care visits, and the proportion of children receiving BCG and measles vaccines. Regarding age standardization when reporting health care use rates, we cannot reliably age adjust these figures as we only collected data from those aged 0 to 5 and 15 to 49. Though the research team considered assessing the factors strongly associated with the update and utilization of services at the household level via multivariate regression, we opted to reserve the reporting of coefficients for subsequent papers we intend to generate as part of this body of work.

#### Barriers to receiving high quality care

The research team collected information about barriers to receiving high quality care in the household survey. For patients who had sought care in the previous 12 months, we estimated the proportion who experienced a range of problems with the care provided, such as poor-quality services, patient experience, and lack of services or supplies. For patients who did not seek care for severe acute illness, we summarized the reasons for not seeking care. Respondents could report multiple reasons and problems. All analyses were conducted at the catchment level.

#### Healthcare spending and affordability

The research team sought to replicate key metrics produced by the WHO’s Global Health Observatory in order to contextualize healthcare spending among survey respondents [21]. We estimated the rates of catastrophic health expenditure incurred by participating households as the proportion of households whose expenditures on healthcare are more than 10% or 25% of their total household expenditure. Within each country, we determined the household per person monthly expenditure and estimated the association between one third of expenditure and consultation rates, number of ANC visits among pregnant women, and receipt of BCG vaccine. Specifically, households were grouped into three income categories: bottom third, middle third, and top third. Models were adjusted for age and sex. We also conducted an analysis of the odds of seeking care by tertile of household expenditure. Furthermore, we opted to collect expenditure data by asking household heads to recall their monthly expenses across a series of categories. Our CHE definitions and data collection methods have been externally validated by several studies conducted in similar research contexts [22–24].

#### Facility readiness and availability

We report on the average readiness and availability percentiles across surveyed facilities in Liberia and Sierra Leone. Service availability is described by an index using a set of ‘tracer’ indicators, which “aim to provide objective information about whether or not a facility meets the required conditions to support the provision of basic or specific services with a consistent level of quality and quantity” [25]. Service readiness is described using the five general service readiness domains defined by the SARA tool (basic amenities, basic equipment, diagnostic equipment, standard precautions, essential medicines, and general [overall] readiness). A score is generated per domain based on the number of domain elements present at a given facility, and an overall general readiness score is calculated based on the mean of the five domains.

## Results

Overall, we surveyed 1,051 unique households and conducted individual surveys with 1,998 adults and 578 children within those households. Table 1 reports summary statistics of the population and sample. Respondent age and sex were comparable between sites, though larger discrepancies were observed in terms of working status. The research team administered all seven sections of the SARA survey in 11 facilities in Sierra Leone and 4 facilities in Liberia. Refer to S1 Appendix for a summary of core facility characteristics across countries.

**Table 1:** Summary statistics of the samples from the household survey. Values are mean (sd) unless otherwise stated.

		Sierra Leone					Liberia			
		Sewafe	Kombayende	Gandorhun	Kayima	Total	Harper	Karluway-2	Pleebo	Total
**Sample**										
N	Households	103	114	134	136	487	257	36	269	562
	Residents of households	329	388	334	374	1425	526	70	555	1151
	Adults	257	304	263	289	1113	407	54	424	885
	Under-fives	72	84	71	85	312	119	16	131	266
Age, years	Men	30.4 (11.0)	31.3 (11.6)	30.3 (10.0)	31.7 (9.5)	30.9 (10.5)	28.2 (9.7)	28.5 (10.4)	27.9 (9.8)	28.1 (9.8)
	Women	28.0 (8.5)	28.2 (9.1)	29.2 (9.1)	29.4 (8.7)	28.7 (8.9)	29.1 (9.5)	27.7 (8.1)	28.6 (9.2)	28.7 (9.3)
	Under 5	2.6 (1.2)	2.7 (1.0)	2.5 (1.1)	2.6 (1.2)	3.4 (5.2)	2.3 (1.3)	2.3 (1.3)	2.2 (1.1)	2.2 (1.2)
Sex, male %	Adult	46	45	47	45	46	48	44	45	47
	Under 5	57	53	35	50	49	51	19	50	49
Education, highest level attended % (adults)	Primary/pre-primary	17	16	18	10	15	21	44	19	21
	Secondary	40	24	33	14	27	34	15	45	38
	Tertiary	3	2	2	1	2	22	4	12	16
Currently working %	Men	64	69	63	79	69	35	17	31	32
	Women	48	70	66	80	64	18	10	13	15

### Care seeking and utilization

**Fig 2:**
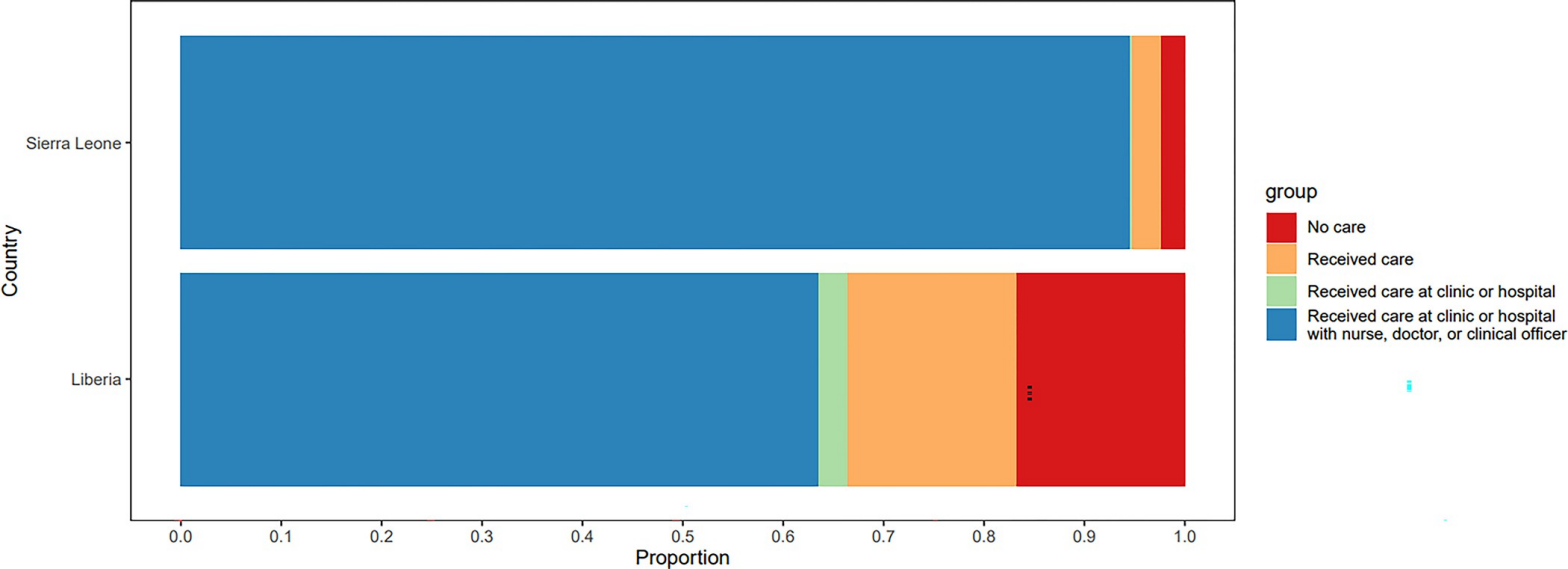
Proportion of respondents seeking treatment, receiving care at a facility, and receiving care from a trained healthcare provider.

In terms of demand for health care, 83% of patients reported any acute illness across implementation sites in the preceding month, and 49% reported experiencing severe acute illness. Fig 2 below displays the proportion of respondents in our sample who sought care for severe acute illness symptoms. The propensity to receive care when ill appears higher in Sierra Leone than Liberia, with approximately 90% of respondents seeking treatment compared to roughly 70% in Maryland County. Only 63% of respondents in Liberia with severe symptoms received care with a clinical professional in a clinic or hospital.

Table 2 reports several care use statistics. Outpatient consultation rates showed little variation across catchment areas within sites. The mean consultation rate across sites was 2.8 and 1.5 outpatient visits per person-year in Sierra Leone and Liberia, respectively. In both settings, children under five reported more visits per year overall. ANC use among pregnant women was between 85% and 95% in all sites. Only a minority of children under five had received BCG or 1^st^ or 2^nd^ doses of measles vaccinations. We report vaccination uptake as an indicator for healthcare utilization for children under five.

**Table 2:** Health care use rates, by site and catchment area. Values are percentages [95% confidence interval].

	Sierra Leone					Liberia			
	Sewafe	Kombayende	Gandorhun	Kayima	Total	Harper	Karluway-2	Pleebo	Total
Outpatient consultation rate, adults (visits per person-year)	3.1 [0.0, 6.3]	2.8 [0.0, 6.1]	2.7 [0.0, 5.8]	2.4 [0.0, 5.9]	2.8 [0.0, 6.1]	1.5 [0.0, 5.1]	2.0 [0.0, 6.7]	1.5 [0.0, 4.8]	1.5 [0.0, 4.3]
Inpatient discharge rate, adults (visits per person-year)	0.6 [0.0, 3.3]	0.2 [0.0, 1.6]	0.3 [0.0, 1.8]	0.3 [0.0, 1.7]	0.4 [0.0, 2.7]	0.7 [0.0, 4.3]	0.5 [0.0, 3.6]	0.3 [0.0, 2.4]	0.5 [0.0, 3.4]
Women with at least 4 ANC visits (%)	93 [88, 98]	90 [83, 97]	89 [82, 96]	89 [83, 96]	91 [88, 94]	82 [68, 96]	80 [44, 100]	86 [75, 97]	85 [77, 93]
Under 5s received BCG vaccine (%)	49 [37, 61]	32 [21, 43]	42 [29, 55]	39 [28, 50]	41 [35, 47]	48 [39, 57]	51 [27, 75]	50 [41, 59]	49 [43, 55]
Under 5s received 1st dose of measles vaccine	41 [29, 53]	49 [37, 61]	64 [52, 76]	62 [51, 73]	52 [46, 58]	37 [28, 46]	46 [21, 71]	40 [31, 49]	39 [33, 45]
Under 5s received 2^nd^ dose of measles vaccine	21 [11, 31]	33 [22, 44]	38 [26, 50]	32 [22, 42]	30 [25, 35]	18 [11, 25]	37 [12, 62]	22 [15, 29]	21 [16, 26]

Based on our healthcare utilization findings, we know that patients seek care from a variety of informal sources. We intended to capture this information in each iteration of individual survey questions asked after a respondent indicated they sought treatment, and the provider choice options included in the instrument were as follows: medical doctor, nurse, midwife, community health officer (CHO), dentist, traditional practitioner, and other. In both country contexts, nurses were most frequently visited, followed by doctors, though Liberia and Sierra Leone site teams have confirmed that it is likely that patients conflate any provider they visit at a facility as a doctor, so the prevalence of visits to doctors is likely overstated within our sample. Few patients were seen by midwives, CHOs, or dentists, and even fewer reported seeking care from traditional medicine practitioners. Both site teams acknowledge that the proportion of respondents who cited receiving care from a traditional healer is likely underreported, given the social desirability bias that frequently accompanies care seeking in Kono District and Maryland County (individuals are more likely to report being visited by a trained provider when being surveyed). The most frequently cited ‘other’ response were pharmacists/drug peddlers, who often fill gaps in care in both Maryland County and Kono District.

#### Barriers to care

We also investigated commonly cited problems with care among respondents who did seek treatment when ill or injured. Results are detailed in Table 3 below. Notably, 84% of respondents who did not seek care when experiencing acute illness in Sierra Leone cited an inability to afford their visit as a barrier to care, while this proportion is much lower in Liberia at 23%. Commonly cited problems with care across countries included long wait times at hospitals and clinics, and lack of essential drugs/equipment.

**Table 3:** Reasons for not seeking care among those with severe acute illness symptoms, and problems with care reported by those who attended visits. Values are percentages [95% confidence interval].

	Sierra Leone					Liberia			
	Sewafe	Kombayende	Gandorhun	Kayima	Total	Harper	Karluway-2	Pleebo	Total
Reasons for not seeking care among those with severe acute illness									
n	21	19	10	8	58	36	7	38	81
Inability to afford visit	81% [64, 98]	89% [75, 100]	70% [52, 98]	10% [0, 31]	84% [75, 93]	14% [3, 25]	43% [6, 80]	29% [15, 43]	23% [12, 35]
Difficulty reaching facility	19% [2, 36]	32% [11, 53]	20% [0, 45]	13% [0, 36]	22% [11, 33]	22% [8, 36]	43% [6, 80]	26% [12, 40]	26% [16, 26]
Experienced issues with providers’ services	0% [0, 16]	5% [0, 15]	10% [0, 29]	0% [0, 37]	3% [0, 7]	17% [5, 29]	0% [0, 41]	11% [1, 21]	12% [5, 19]
Other	19% [2, 36]	21% [3, 39]	30% [2, 58]	25% [0, 55]	22% [11, 33]	53% [37, 69]	43% [6, 80]	53% [37, 69]	52% [41, 63]
Problems with care among those who received care for acute illness									
n	194	123	135	99	551	205	28	175	549
Waiting	39% [32, 46]	37% [28, 46]	20% [13, 27]	32% [23, 41]	32% [28, 36]	58% [51, 65]	57% [39, 75]	45% [38, 52]	39% [35, 43]
Cost	11% [7, 15]	20% [13, 27]	11% [6, 16]	11% [5, 17]	13% [10, 16]	4% [1, 7]	11% [0, 22]	4% [1, 7]	3% [2, 4]
Poor quality care	18% [13, 23]	13% [7, 19]	9% [4, 13]	15% [8, 22]	14% [11, 17]	12% [8, 16]	21% [6, 36]	11% [6, 16]	9% [7, 11]
Lack of drugs/ equipment	26% [20, 32]	46% [37, 55]	38% [30, 46]	31% [22, 40]	34% [30, 38]	16% [11, 21]	25% [9, 44]	18% [12, 24]	13% [10, 16]
Other	7% [3, 11]	4% [1, 7]	4% [1, 7]	6% [1, 11]	5% [3, 7]	5% [2, 8]	14% [1, 27]	6% [2, 10]	5% [3, 7]

#### Health care spending

**Fig 3:**
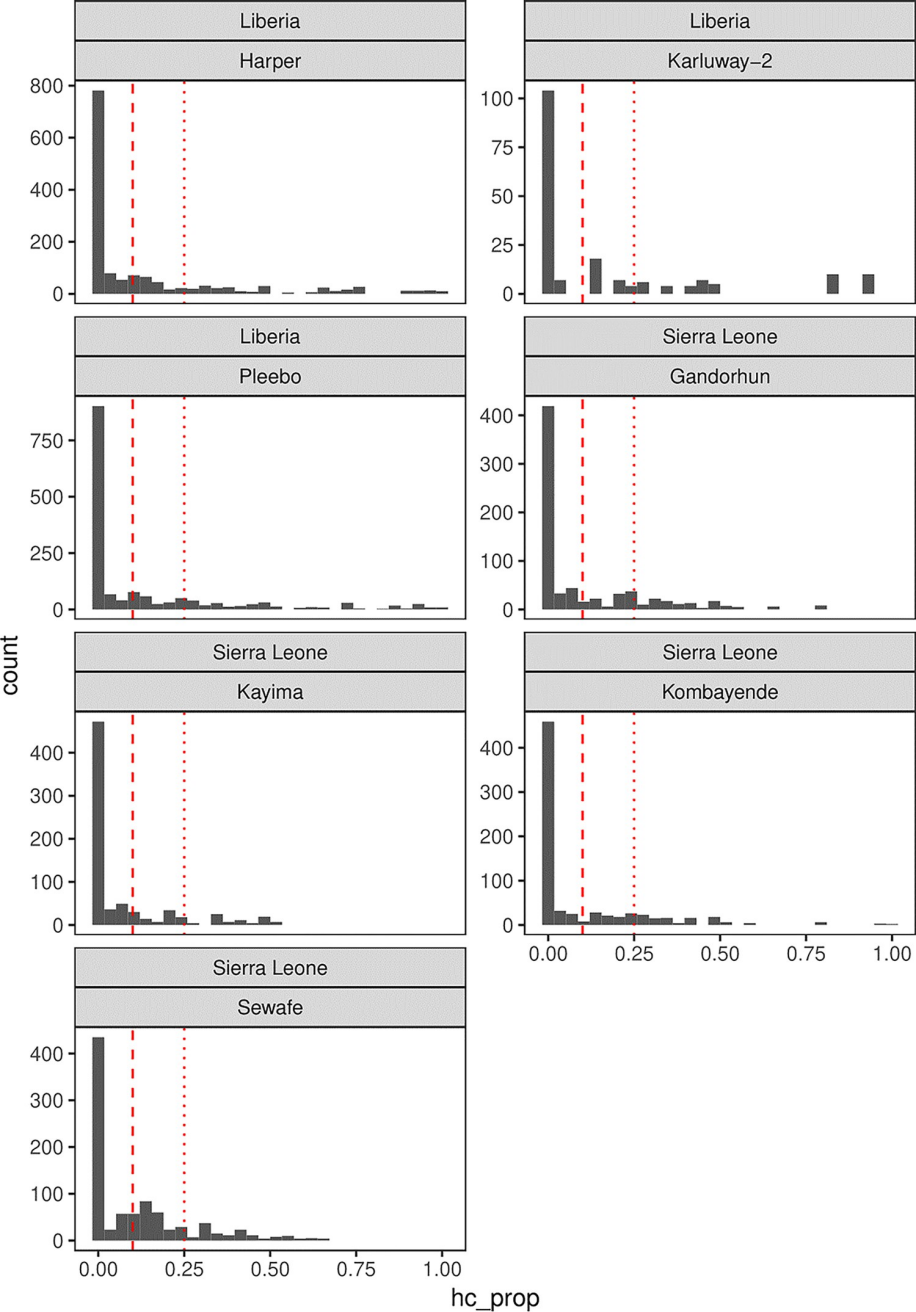
Proportion of total household budget spent on healthcare in the preceding month by catchment area with red lines indicating 10% (dashed) and 25% (dotted) of total expenditure.

Fig 3 reports the proportion of total household budget spent on health care in the preceding month, by catchment area. As demonstrated in the visualization, in Kono, Sierra Leone, we estimated that 30% of households spent greater than 10% of their total expenditure on healthcare in a month, and that 15% of households spent greater than 25%. In our Liberia sample population, these healthcare expenditure figures were 32% and 14%, respectively. Contextually, it is worth reporting the income levels reported by households in our sample. We collected income data via a multiple choice question that detailed monthly income buckets ranging from less than 3000 LRD/150,000 LE (18 USD/7 USD) to 40,000 LRD/2,000,000 LE (245/102 USD). In Kono District, 41% of respondents reported that their household makes less than 150,000 LE per month (the lowest income category recorded on our questionnaire). By contrast, in Maryland County, 21% of households report total monthly incomes below 3,000 LRD (50% of households report incomes between 3,000 and 8,900 LRD (these comprise the three lowest income categories recorded on our questionnaire).

**Fig 4:**
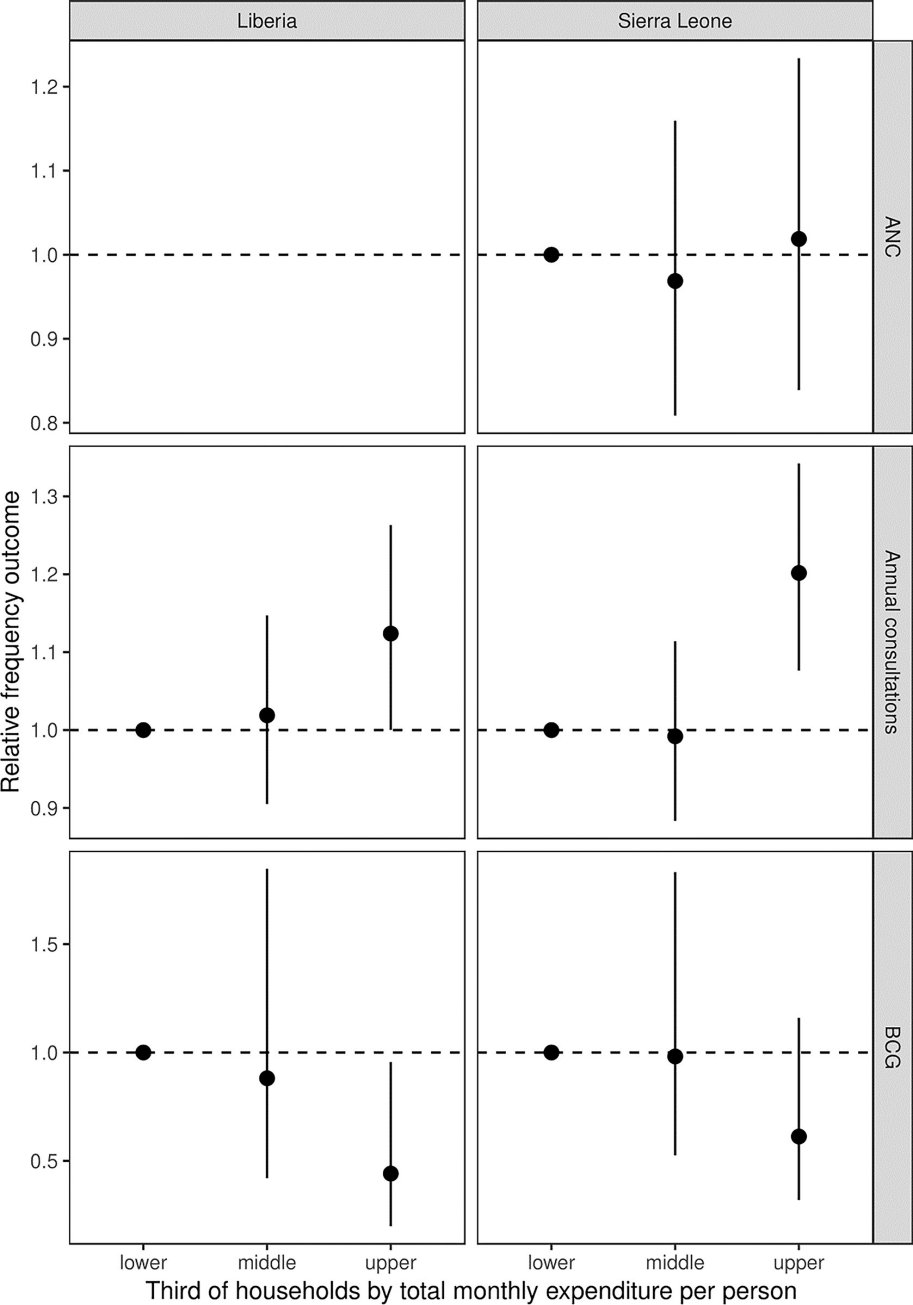
Odds ratios for care use associated with level of total households per person expenditure.

Fig 4 displays the odds of seeking care for acute illnesses by third of households according to total monthly household expenditure per person adjusted for age and sex. Across sites, the adjusted consultation rate was 10% to 20% higher among the top third of households. There was little evidence for differences for ANC. Higher spending households appeared to have lower probability of BCG.

### Facility service availability and readiness

In terms of service availability, 100% of facilities in Sierra Leone and Liberia offered basic obstetric and newborn care, antenatal care, routine immunization services, and one or more child preventative or curative services. 75% and 91% of facilities in Liberia and Sierra Leone respectively offered tuberculosis services–the exceptions were Boniken Health Center in Liberia, and Kangama CHC in Sierra Leone. Only two of the four facilities in Liberia offered basic non-communicable disease (NCD) services, along with six of 11 facilities in Sierra Leone. Per SARA guidelines, NCD services include management and diagnosis of diabetes, cardiovascular disease, chronic respiratory disease, and cervical cancer; facilities included in our sample showed a particularly apparent gap in the availability of services for cervical cancer.

**Fig 5:**
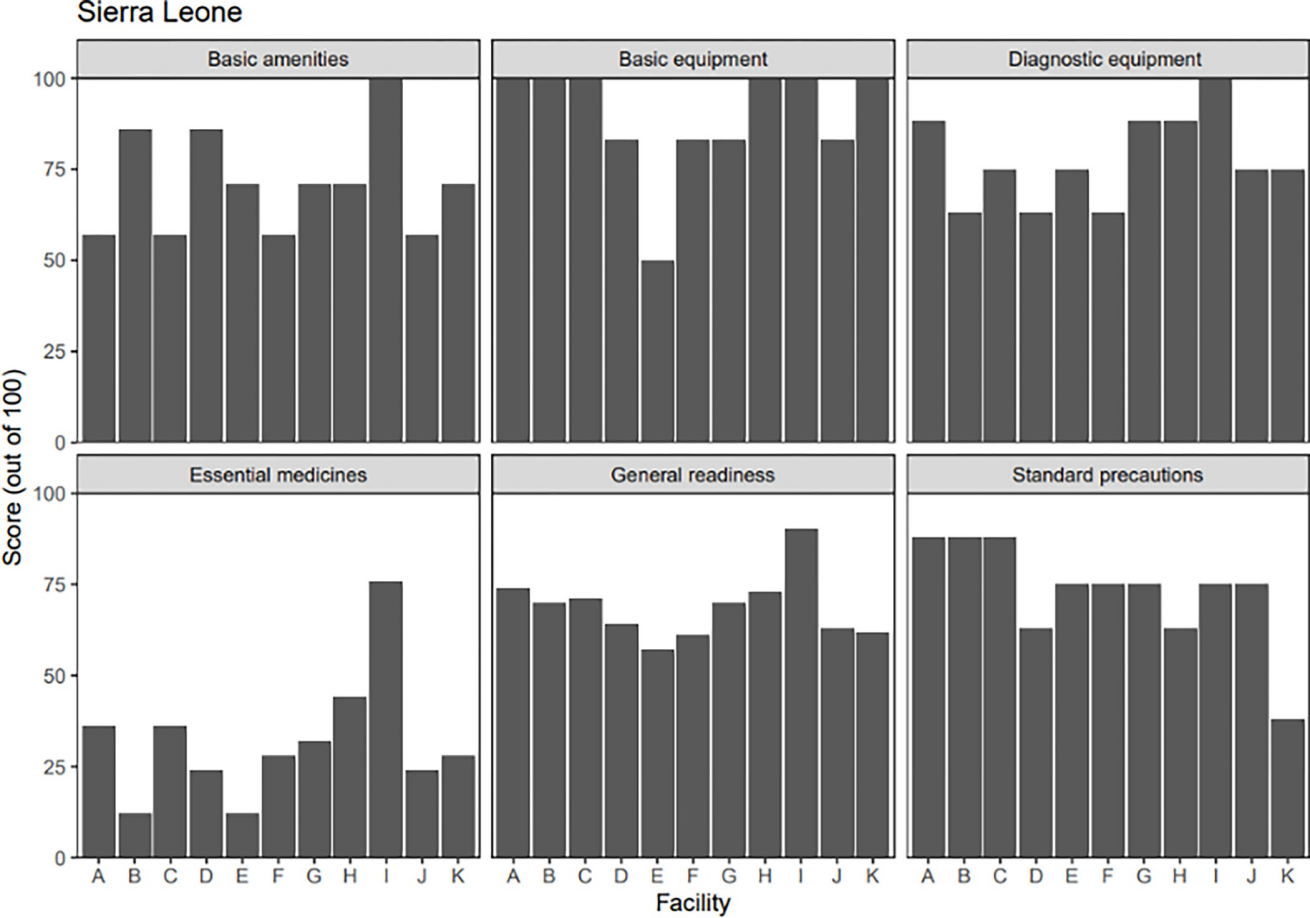
SARA survey readiness score summary, by facility, in Sierra Leone. Legend of facilities: A–Gandorhun, B–Kainkordu CHC, C–Kangama CHC, Kayima CHC, Kombayende CHC, F–Sewafe, G–Tombodu CHC, H–UMC Clinic, I–Wellbody Clinic, J–Yengema Clinic, K–Yormandu CHC.

**Fig 6:**
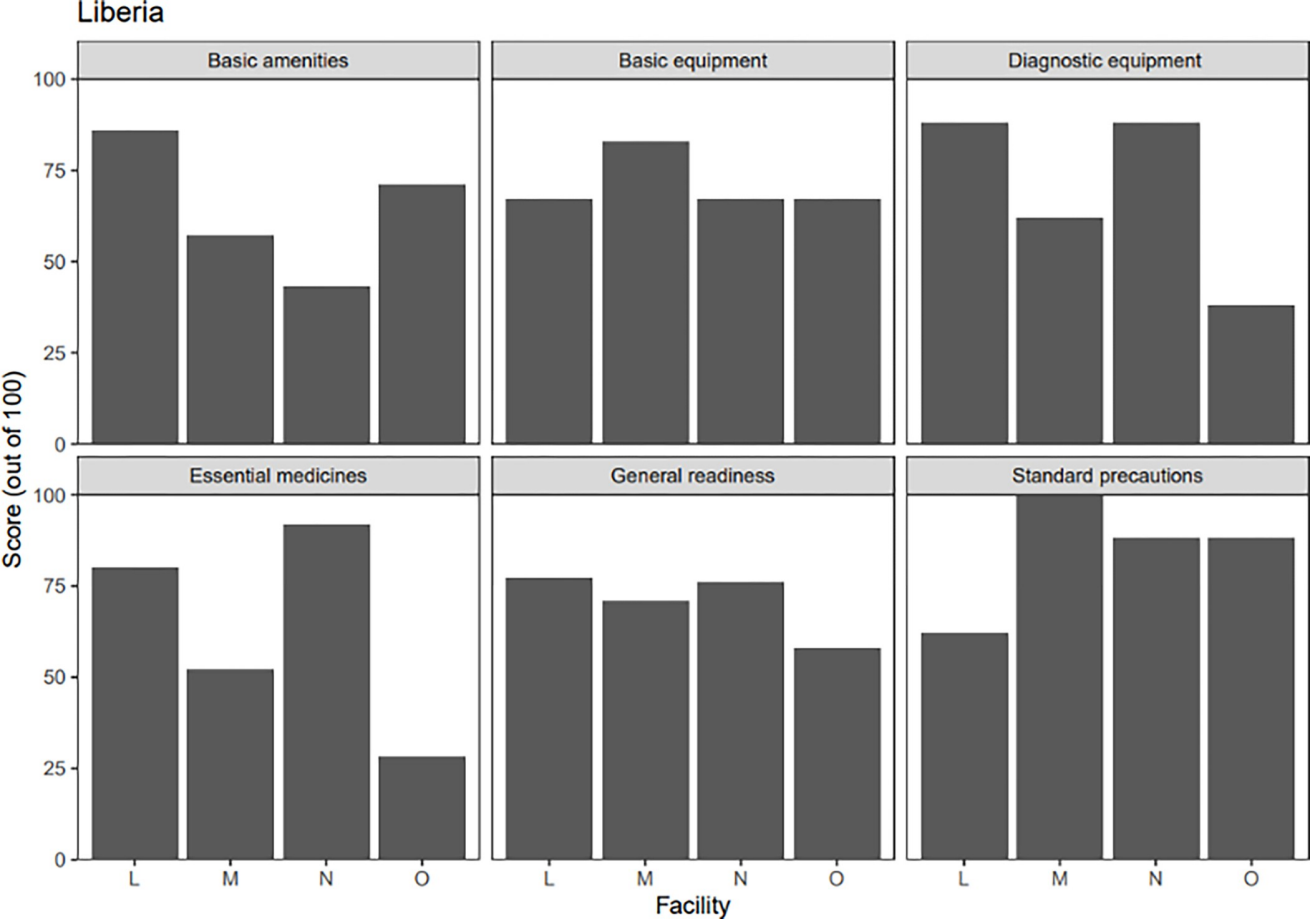
SARA survey readiness score summary, by facility, in Liberia. Legend of facilities: L–Pleebo Health Center, M–Karloken (Edith Wallace) Health Center, N–JJ Dossen Hospital, O–Boniken Health Center.

Figs 5 and 6 below summarize the average readiness score across catchment areas in Kono District and Maryland County. Overall, the general service readiness index mean score for all health centers was similar in both research contexts (70% in Sierra Leone and 71% in Liberia). The greatest hindrance to service readiness was the availability of essential medicines at the health facilities, with facilities in Sierra Leone reporting an average score of 32% and facilities in Liberia reporting a score of 63%. This is consistent with findings from the household survey. For instance, in Sierra Leone, when asked about problems with accessing care, 41% of respondents noted the lack of medication, compared to the 15% of respondents in Liberia who noted this area as a problem with their visit.

## Discussion

This study aimed to identify barriers to achieving universal health coverage in health facility catchment areas in Liberia and Sierra Leone. Overall, this study’s findings serve to complement nationally reported UHC statistics in both country contexts. When coupled with facility readiness and availability scores, experiences and perceptions shared by household survey respondents paint a holistic picture of healthcare use and availability in our surveyed catchment areas. Providing a detailed snapshot of UHC barriers in rural, localized contexts allows us to chart a roadmap for removing them.

### Modifiable barriers to care

#### Barriers to be addressed at the local-clinic level

Previous evidence has illustrated that stockouts of essential medicines often present a barrier to UHC in low-income settings. A 2021 study found that in a survey of 157 pharmacies across seven developing countries, the majority of facilities lacked basic equipment such as thermometers or scales [26]. A related paper identifies weak health product supply chains as the root cause of in many developing countries [27]. Our study’s results were consistent with these findings and highlighted that many facilities lacked the ability to provide basic equipment or essential medicines, and that a substantial number of household survey respondents cited lack of medication as a problem with recent facility visits. This was particularly true in Kono District, where the proportion of respondents citing lack of essential medicines (41%) as a persisting challenge was significantly higher than that reported by Maryland County participants (15%). The heterogeneity of issues in medication availability across implementation sites suggests that stockout challenges are not insurmountable, and the research team suggests investing in supply chain strengthening efforts to address these issues in PIH catchment areas. Specifically, PIH facilities could benefit from right-sizing forecasting efforts and focusing on improving procurement systems to ensure timely delivery of essential medications in order to prevent medication stockouts.

Additionally, our study’s findings also revealed lack of service availability as a barrier to UHC across implementation sites. NCD services, including care for patients diagnosed with diabetes, cardiovascular disease, chronic respiratory disease, and, most urgently, cervical cancer, emerged as specific areas of concern in facilities where the SARA survey was administered. In the Sierra Leone context, the particularly low readiness score for NCD services is likely driven by two low-performing health centers, and the fact that a larger population of individuals reside in catchment areas with poor scoring facilities. The research team anticipates that direct investments in NCD services across facilities will incentivize increased healthcare utilization among our survey respondents and increase average readiness scores in future SARA assessments. External studies have posited that low uptake of core immunizations (BCG, measles) is linked to the unavailability of vaccination services at under-resourced clinics [28]. However, given that all surveyed facilities reported SARA service availability scores of 100% for immunizations, more plausible explanations for low uptake among children under 5 in our study’s sample include low stocks of vaccines, or lack of funds or transport preventing parents from traveling to facilities for their children’s inoculations. It is worth noting that NCD services are not currently included in free health service programs in either Kono District or Maryland County; working with local governments to advocate for inclusion of NCD care in legislation that subsidizes treatment in other clinical areas could significantly improve health care utilization and patient outcomes.

#### Barriers to be addressed by households and community members

In terms of demand for health care in our research settings, 83% of patients report acute illness across implementation sites, yet consultation rates remain low, especially when compared to Organization for Economic Cooperation and Development (OECD) country rates. For instance, the median number of outpatient visits for all people in countries that are members of the OECD was six to seven visits per person-year [29]. In the United States, the figure is just over three visits annually. A similar study of people who live in slums reports a rate of 0.5 visits per person-year for the urban poor in Lagos and Ibadan, Nigeria, and a rate of 1.5 visits for those in slums in Nairobi, Kenya [10]. The latter is comparable to the 1.5 outpatient visits we observed in Maryland County and 2.8 visits in Kono District. Additionally, the observed drop-off when analyzing the cascade of respondents who express symptoms, to those who seek facility-based care, to those who are treated by a trained healthcare provider, is also observed in similar research contexts [30–32]. While the responsibility for seeking treatment when ill ultimately lies with households and community members, it is imperative that the Liberian and Sierra Leonean governments provide quality services in order to incentivize care seeking. Facility-level improvements that could be made to target low utilization could include comprehensive treatment availability and increased staffing and diagnostic capacities.

#### Barriers to be addressed through governmental action

External results from studies conducted in similar research contexts cite high visit costs as a primary deterrent from seeking medical care [30, 33, 34]. Respondents in our sample reported cost of care as a barrier to healthcare utilization: inability to afford visits costs was a commonly cited reason for not seeking care among 84% of household members in Kono District and 23% of household members in Maryland County. Additionally, a subset of respondents across implementation sites who did seek care when needed cited visit cost as a persisting problem. To address this challenge, the research team suggests introducing strategies to reduce the cost of health services across implementation sites. By removing associated costs, we anticipate more people will use the health services whenever they need them, and recommend that policymakers remove user fees or find ways to subsidize the cost of health care across facilities. Further, this research underscores the critical need to increase current health expenditure as a percentage of gross domestic product (GDP); in countries with low GDPs, even high current health expenditure is inadequate. Ensuring free or subsidized visit costs for all patients, regardless of age, gender, or disease profile, would require close collaboration with government partners, namely, the Sierra Leonean and Liberian Ministries of Health. In settings where care is already free or subsidized, governmental or third party monitoring at the subnational or local level is imperative to ensuring compliance with policies that make treatment accessible and affordable.

Furthermore, our study’s analyses of catastrophic health expenditure show that over 30% of households in both Liberia and Sierra Leone spent greater than 10% of their total monthly expenditure on healthcare. Households in Kono District appeared to bear the weight of high health care costs more acutely than their counterparts in Maryland County, which warrants further investigation. In Liberia, user fees were suspended at the primary care level for all services included in the Basic Package of Health Services in 2007 [35]. In our findings, a lower percentage of Liberia households reported cost as a barrier to care compared to surveyed households in Sierra Leone, which may be related to the suspension of user fees at public governmental facilities in Liberia. This suggests that engaging with the Ministry of Health in Sierra Leone to advocate for the adoption of similar legislation would help target the barriers to care that persist in the country.

Overall, we found that respondents continue to incur indirect and informal costs associated with medical visits, including services in ‘free’ health care categories. The research team acknowledges that further investigation is required to understand the reasons for persisting user fees for patients who should be receiving free healthcare services, and in the interim, recommends that subsidies are scaled up to include coverage for costs of additional expenditures associated with facility visits, such as medication and transportation fees. External evidence suggests that, given the choice, patients may be willing to pay more to receive higher quality or more accessible care; a 2021 study in slum settings estimated a positive willingness to pay for 15 minutes less of travel time to a health facility [36].

#### Non-modifiable barriers to care

Though the research team acknowledges the difficultly of completely removing barriers to care in rural Liberia and Sierra Leone, we do not view any of these challenges as insurmountable. Transportation barriers can be categorized as a non-modifiable barrier to care in the intermediate term, given that this challenge is directly related to facilities’ physical locations being far away from rural households as well as quality of roads and other transportation infrastructure.

#### Well-performing clinical areas

In addition to identifying gaps in service provision, this study’s results illustrated progress towards UHC in the context of maternal and child health. Our findings demonstrate that across sites, 83% of pregnant women received at least four antenatal care visits, which is consistent with findings at the national level for both countries (79% in Sierra Leone and 87% in Liberia). High prenatal visit rates reported at the household level are aligned with the high service availability scores reported for antenatal, obstetric, and newborn care reported by facilities, which bolsters the argument for increasing service availability to meet patient demand. The strong performance of these metrics may be attributable to the programmatic focus on maternal health at community health centers that are affiliated with PIH, though this hypothesis warrants further investigation. Furthermore, Sierra Leone introduced a free healthcare initiative in 2010 aimed at reducing medical expenses for women and children, which may be correlated with higher utilization rates for MCH services, compared to other clinical areas.

#### Study limitations

This study’s findings should be interpreted alongside considerations of its limitations. Firstly, the household and SARA surveys were conducted in rural areas of Liberia and Sierra Leone, and findings are not nationally representative (by stating this, we mean that our sample sizes are not sufficiently powered to imply that these findings would be replicated throughout all of Sierra Leone and Liberia, though this does not preclude us from suggesting policy solutions based on the findings documented in this paper). While our primary objective was to achieve a local understanding of barriers to care, we believe these results to be a useful exemplar of UHC analysis, rather than providing generalizable findings. Second, in March 2020 the SARS-CoV-2 pandemic halted survey work in Liberia and so only approximately half of the intended households were surveyed limiting our inferences in these areas. The pandemic may have also led to large changes in patient attitudes and health system capacity that might alter the nature of the barriers to care faced by local residents. As a result, subsequent rounds of these surveys are planned. Third, our interpretation of the results and proposal of solutions is based on expert opinion rather than confirmatory studies. For example, lack of vaccinations may result predominantly from hesitancy rather than poor supply chains, although we do not believe this to be the case. We will be evaluating changes in the metrics associated with the planned interventions in follow-up studies.

## Conclusion

Our findings suggest that the cost of care remains a major barrier to care seeking among household and SARA survey respondents. While the propensity to seek care when ill is high in both Maryland County and Kono District, a considerable proportion of households continue to spend a significant portion of their total expenditure on healthcare. The availability of essential medicines and equipment in health facilities is limited, which poses a risk to the delivery of quality care. To address these barriers and improve access to healthcare services, the research team recommends the implementation of targeted interventions, including visit cost subsidies and supply chain improvements. These interventions would help to reduce the financial burden of healthcare expenses and ensure the availability of essential medicines and equipment.

In conclusion, this study provides valuable insights into the challenges faced by healthcare systems in PIH’s catchments areas, and offers practical solutions to address them. By implementing the recommended interventions, facilities and households can make significant progress towards achieving universal health coverage and ensuring that rural communities receive the health services they need without suffering financial hardship.

## Supporting information

S1 Checklist PLOS questionnaire provides our responses to PLOS’ questionnaire on inclusivity in global research. (DOCX)

S1 Data Provides access to the household survey data and code used for analysis for both countries. (ZIP)

S2 Data Provides access to the services availability and readiness assessment (SARA) data and code used for analysis for both countries. (ZIP)

S1 Appendix Household survey mapping, intervention status, facility type, and patient care summary for all facilities included in SARA survey (data collected by PIH in activities external to this research). (DOCX)

S2 Appendix Household survey instruments. (DOCX)

S3 Appendix SARA survey instrument. (XLSX)
