# Supplementary material for: Community and facility-level barriers to achieving UHC in Kono District, Sierra Leone and Maryland County, Liberia
Source: PLOS Glob Public Health. 2023 Jun 26;3(6):e0002045. doi: 10.1371/journal.pgph.0002045 (PMC10292700; doi:10.1371/journal.pgph.0002045)
Supplement: S1 Appendix — (DOCX) [file pgph.0002045.s004.docx]

| **Country Site** | **Facility** | **Associated household survey catchment area, if relevant** | **Intervention Status** | **Start of PIH support** | **Facility Type** | **Facility type (primary, secondary, tertiary)** | **User fee** | **Specific services offered** |
| --- | --- | --- | --- | --- | --- | --- | --- | --- |
| Liberia | Boniken Clinic |  | PIH-supported site | 2019 | Clinic | Primary | Free | Maternity & Delivery, Family planning, ANC, General OPD, Lab, Dispensary, Postnatal Immunization, Malnutrition |
|  | Edith Wallace Health Center |  | PIH-supported site | 2020 | Health Center | Primary | Free | Triage & Screening, General OPD, Lab, Dispensary, ANC, Maternity & Delivery, Family Planning, Postnatal, Immunization, malnutrition, HIV, TB, Mental Health, NCD |
|  | JJ Dossen Memorial Hospital |  | PIH-supported site | 2015 | Hospital | Tertiary | Free | Triage & Screening, General OPD, Inpatient Care, Surgery, Emergency Unit, Pediatrics NICU, Malnutrition, Lab, ANC, Maternity & Delivery, Family Planning, Postnatal, Immunization, malnutrition, HIV_ART, TB, MDR_TB, Mental Health, NCD, Pharmacy, Kitchen, Palliative Care, Eye Care, Biomedical (oxygen production), KMC, |
|  | Pleebo Health Center | Pleebo | PIH-supported site | 2015 | Health Center | Secondary | Free | Triage & Screening, General OPD, Lab, ANC, Maternity & Delivery, Family Planning, Postnatal, Immunization, Malnutrition, HIV, TB, Mental Health, NCD, KMC, Kitchen, Emergency Care, Pharmacy, Palliative Care |
| Sierra Leone | Koidu Government Hospital (KGH) | Patients from multiple catchment areas utilize this facility | PIH-supported site | 2015 | Hospital | Secondary | Base rate of $1 USD (at time of survey)/10,000 Leones  Government policy: Government of SL required fee for all services, excluding those for under-5 (under-5 includes maternal care) | ANC, HIV, TB, General OPD, Inpatient, Lab, Pharmacy, Maternity & Delivery, Triage & Screening, Mental Health, Immunization, Malnutrition, Observation, Postnatal, surgery, emergency unit, AFYS, Family planning |
|  | Kombayende Community Health Centre | Kombayende | Control site | 2021 | Health Center | Primary | Base rate of $1 USD (at time of survey)/10,000 Leones  Government policy: Government of SL required fee for all services, excluding those for under-5 (under-5 includes maternal care) | ANC, HIV, TB, General OPD, Lab, Pharmacy, Maternity & Delivery, Triage & Screening, Mental Health, Immunization, Malnutrition, Observation, Postnatal and, Family planning |
|  | Sewafe Clinic | Sewafe | Control site | 2019 | Health Center | Primary | Base rate of $1 USD (at time of survey)/10,000 Leones  Government policy: Government of SL required fee for all services, excluding those for under-5 (under-5 includes maternal care) | ANC, HIV, TB, General OPD, Lab, Pharmacy, Maternity & Delivery, Triage & Screening, Mental Health, Immunization, Malnutrition, Observation, Postnatal and, Family planning |
|  | Wellbody Clinic | Patients from multiple catchment areas utilize this facility | PIH-supported site | 2015 | Health Center | Primary | Base rate of $1 USD (at time of survey)/10,000 Leones  Government policy: Government of SL required fee for all services, excluding those for under-5 (under-5 includes maternal care) | ANC, HIV, TB, General OPD, Lab, Pharmacy, Maternity & Delivery, Triage & Screening, Mental Health, Immunization, Malnutrition, Observation, Postnatal and, Family planning |
|  | Gandorhun | Gandorhun | Control site |  | Health Center | Primary | Base rate of $1 USD (at time of survey)/10,000 Leones  Government policy: Government of SL required fee for all services, excluding those for under-5 (under-5 includes maternal care) | ANC, HIV, TB, General OPD, Lab, Pharmacy, Maternity & Delivery, Triage & Screening, Mental Health, Immunization, Malnutrition, Observation, Postnatal and, Family planning |
|  | Kainkordu CHC | Patients from multiple catchment areas utilize this facility | Control site |  | Health Center | Primary | Base rate of $1 USD (at time of survey)/10,000 Leones  Government policy: Government of SL required fee for all services, excluding those for under-5 (under-5 includes maternal care) | ANC, HIV, TB, General OPD, Lab, Pharmacy, Maternity & Delivery, Triage & Screening, Mental Health, Immunization, Malnutrition, Observation, Postnatal and, Family planning |
|  | Kangama CHC | Patients from multiple catchment areas utilize this facility | Control site |  | Health Center | Primary | Base rate of $1 USD (at time of survey)/10,000 Leones  Government policy: Government of SL required fee for all services, excluding those for under-5 (under-5 includes maternal care) | ANC, HIV, TB, General OPD, Lab, Pharmacy, Maternity & Delivery, Triage & Screening, Mental Health, Immunization, Malnutrition, Observation, Postnatal and, Family planning |
|  | Kayima CHC | Kayima | Control site |  | Health Center | Primary | Base rate of $1 USD (at time of survey)/10,000 Leones  Government policy: Government of SL required fee for all services, excluding those for under-5 (under-5 includes maternal care) | ANC, HIV, TB, General OPD, Lab, Pharmacy, Maternity & Delivery, Triage & Screening, Mental Health, Immunization, Malnutrition, Observation, Postnatal and, Family planning |
|  | Tombodu CHC | Patients from multiple catchment areas utilize this facility | Control site |  | Health Center | Primary | Base rate of $1 USD (at time of survey)/10,000 Leones  Government policy: Government of SL required fee for all services, excluding those for under-5 (under-5 includes maternal care) | ANC, HIV, TB, General OPD, Lab, Pharmacy, Maternity & Delivery, Triage & Screening, Mental Health, Immunization, Malnutrition, Observation, Postnatal and, Family planning |
|  | UMC Clinic | Patients from multiple catchment areas utilize this facility | Control site |  | Clinic | Primary | Base rate of $1 USD (at time of survey)/10,000 Leones  Government policy: Government of SL required fee for all services, excluding those for under-5 (under-5 includes maternal care) | ANC, HIV, TB, General OPD, Lab, Pharmacy, Maternity & Delivery, Triage & Screening, Mental Health, Immunization, Malnutrition, Observation, Postnatal and, Family planning |
|  | Yengema CHC | Patients from multiple catchment areas utilize this facility | Control site |  | Health Center | Primary | Base rate of $1 USD (at time of survey)/10,000 Leones  Government policy: Government of SL required fee for all services, excluding those for under-5 (under-5 includes maternal care) | ANC, HIV, TB, General OPD, Lab, Pharmacy, Maternity & Delivery, Triage & Screening, Mental Health, Immunization, Malnutrition, Observation, Postnatal and, Family planning |
|  | Yormandu CHC | Patients from multiple catchment areas utilize this facility | Control site |  | Health Center | Primary | Base rate of $1 USD (at time of survey)/10,000 Leones  Government policy: Government of SL required fee for all services, excluding those for under-5 (under-5 includes maternal care) | ANC, HIV, TB, General OPD, Lab, Pharmacy, Maternity & Delivery, Triage & Screening, Mental Health, Immunization, Malnutrition, Observation, Postnatal and, Family planning |
