# Supplementary material for: Community and facility-level barriers to achieving UHC in Kono District, Sierra Leone and Maryland County, Liberia
Source: PLOS Glob Public Health. 2023 Jun 26;3(6):e0002045. doi: 10.1371/journal.pgph.0002045 (PMC10292700; doi:10.1371/journal.pgph.0002045)
Supplement: S2 Appendix — (DOCX) [file pgph.0002045.s005.docx]

**IMPACT INITIATIVE HOUSEHOLD SURVEY**

# MODULE 6: CHILDREN’S QUESTIONNAIRE

**VERSION 3 05/09/19**

X: INTERVIEWER DETAILS

| X1 | Interviewer name | [Drop down list of interviewers] |  |
| --- | --- | --- | --- |
| X2 | Village name | [Drop down list of villages] |  |
| X3 | Segment ID | [ID number of the segment] |  |
| X4 | Name of child | [Text] |  |
| X4B | Name of respondent (adult) | [Text] |  |
| X5 | Geocode | [Capture GPS location] |  |

A: INTERVIEW ATTEMPTS

| A1.1 | Date of interview (1^st^) | [Date entry] |  |
| --- | --- | --- | --- |
| A1.2 | Start time (1^st^) | [Time entry] |  |
| A1.3 | Result of interview (1^st^) | 1. Proceeding  2. Postponed  3. Refused | -> B1  -> A2.1  -> |
| A2.1 | Date of interview (2^nd^) | [Date entry] |  |
| A2.2 | Start time (2^nd^) | [Time entry] |  |
| A2.3 | Result of interview (2^nd^) | 1. Proceeding  2. Postponed  3. Refused | -> B1  -> A3.1  -> |
| A3.1 | Date of interview (3^rd^) | [Date entry] |  |
| A3.2 | Start time (3^rd^) | [Time entry] |  |
| A3.3 | Result of interview (3^rd^) | 1. Proceeding  3. Refused | -> B1  -> |

B: PARTICIPANT INFORMATION AND CONSENT

[TBC]

3: HEALTH CARE USE, ACUTE CONDITIONS

[Survey form will autofill [NAME] with the child’s name captured above in X4]

NOTE 2

We would now like to ask you about recent health conditions members of your household may have experienced recently.

| 3101 | Has [NAME] suffered from any of the following conditions in the last two weeks? [SELECT ALL THAT APPLY] | 1 Diarrhoea  2 Fever  3 Difficulty breathing/coughing  4 Serious injury  5 Pain  6 Skin problem (ulcers/sores/rashes etc)  7 Anxiety/depression/difficulty sleeping  8 Nausea/dizziness/light-headed  9 Appetite problems  10 Fatigue  96 Other (please specify) | [If none selected] -> 1201 |
| --- | --- | --- | --- |
| 3101_spy | Please specify | [Text] | [IF 3101=96] |
| 3102 | Was the child given any of the following since he/she started having the diarrhoea?  [SELECT ALL THAT APPLY] | 1 A fluid made from a special packet  2 A pre-packaged ORS liquid  3 A government-recommended homemade fluid  4 Zinc tablets or syrup | [IF 3101=1] |
| 3103 | At any time during the illness, did [NAME] have blood taken from his/her finger or heel for testing? | 0 No  1 Yes | [ONLY ASK IF 3101=2] |
| 3104 | Did [NAME]’s test show that he/she had malaria? | 0 No  1 Yes | [IF 3103=1] |
| 3105 | **Liberia ONLY**  What kind of medicine [NAME] took? Any other medicine?  SELECT ALL MENTIONED. | 1 New malaria tablet/ACT  2 Chloroquine  3 Country medicine  4 Antibiotics  96 Other (please specify)  98 Don’t know  99 Prefer not to say | [IF 3104=1] |
| 3106 | **Liberia ONLY**  Did [NAME] sleep under mosquito net last night? | 0 No  1 Yes  98 Don’t know  99 Prefer not to say |  |
| 3107 | What was the nature of the injury? | 1 Road traffic accident  2 Fall or other blunt force  3 Poisoning  4 Burn  96 Other (please specify) | [ONLY ASK IF 1101=4]  ->1104  -> 1102_spy |
| 3107_spy | Please specify | [Text] |  |
| 3108 | How serious was the injury? | 1 Fatal  2 Permanent disability (such as blindness, deafness, loss of ability to walk)  3 Requiring hospitalisation of 10 days or more  4 Requiring hospitalisation of less than 10 days  5 Requiring medical care and resulting in missed work or school  6 None of the above | [ONLY ASK IF 1101=4] |
| 3109 | Did you seek any care for [NAME] for this condition? | 0 No  1 Yes |  |
| 3110 | Why did you not seek care for this condition? | 1 Could not afford the cost of the visit  2 No transport available  3 Could not afford the transport  4 Previous bad treatment  5 Could not take time off work or had other commitments  6 The health care provider’s drugs or equipment were inadequate  7 The health care provider’s skills were inadequate  8 Did not know where to go  9 Tried but were denied health care  10 Was not sick enough  11 Was too sick  12 Did not have permission | [IF 3109 = 0]  [Any response] -> 3201 |
| 3111 | Where did you seek care? | **Sierra Leone**  1 Hospital or clinic  2 Drug store  3 Drug peddler  4 Traditional doctor  5 CHW  6 Church yard  7 At home  96 Other (please specify)  **Liberia**  1 Hospital/clinic  2 Drug store  3 Tablet man/black bagger  4 Country doctor  5 CHV  6 Church yard  7 At home  96 Other (please specify) |  |
| 3111_spy | Please specify | [Text] | [IF 3111=96] |
| 3112 | Please name the hospital/clinic | [Dropdown list] | [IF 3111=1] |
| 3113 | About how long did it take you to get there? | [Integer] Hours  [Integer] Minutes  [Autofill 0] |  |
| 3114 | How did you get there?  [SELECT ALL THAT APPLY] | 1 Private vehicle  2 Public transportation  3 Taxicab  4 Ambulance or emergency vehicle  5 Bicycle  6 Motorbike  7 Walked  96 Other |  |
| 3115 | About how long did the visit take once you had arrived? [Including waiting times for consultation and treatment] | [Integer] Days  [Integer] Hours  [Integer] Minutes  [Autofill 0] |  |
| 3116 | How much did you pay overall for [NAME]’s visit? [Including transport, consultation fees, drugs, and any tests or services] [OVERALL NET COST LESS ANY MONEY RECEIVED] | [Integer] Leones/LD |  |
| 3117 | Why did you choose this provider? [SELECT ALL THAT APPLY] | 1 Nearness of the facility  2 Service providers are nice/friendly  3 Good services are available  4 Short waiting times  5 Qualified doctors are available  6 Low fees/low treatment cost  7 Good waiting arrangements  8 Confidentiality is maintained  9 Do not know where else to go  10 Medicine is also available  11 Availability of diagnostic service  12 Recommendation from someone  96 Other (please specify) |  |
| 3118_spy | Please specify | [Text] | [IF 3118=96] |
| 3119 | Were there any problems with the visit or service? [SELECT ALL THAT APPLY] | 1 Waited too long  2 Inadequate explanation about the problem or treatment  3 Lack of privacy from having other see or hear the examination or visit  4 Lack of medicines  5 Opening hours are inconvenient  6 Opening days are inconvenient  7 Facility is not clean  8 Poor treatment from staff  9 High cost for services or treatments |  |

2: VACCINATIONS AND IMMUNISATIONS

| 3201 | Does the child have a card or other document with their vaccinations written down? | 0 No  1 Yes |  |
| --- | --- | --- | --- |
| 3202 | Has the child received any vaccines since birth up to now? | 0 No  1 Yes |  |
| 3203 | Can I see the vaccination card?  [TAKE A PHOTO] | 0 No  1 Yes | [IF 3201 = 1] |
| 3204 | In addition to what is recorded on this document/these documents did the child receive any other vaccinations, including vaccinations received in campaigns or immunization days or child health days? [MARK THESE BELOW] | 0 No  1 Yes |  |

[VACCINATION TABLE FOR BCG (1 dose), ROTA (2 doses), PENTA (3), PCV (3), OPV (3), IPV (1), IPTi (3), Measles (2), Rubella (1), Yellow Fever (1), Vitamin A (1)]

3: HEALTH AND HEALTH HISTORY

| 3301 | Overall, would you say this child’s health is: | 1 Excellent  2 Very good  3 Good  4 Fair  5 Poor |  |
| --- | --- | --- | --- |
| 3302 | Compared to one year ago, how would you rate this child’s health in general now? | 1 Much better than a year ago  2 Somewhat better than a year ago  3 About the same  4 Somewhat worse than one year ago  5 Much worse than one year ago |  |
| 3303 | **Liberia ONLY**  Has [NAME] ever been treated for low weight/too small/dryness? | 0 No  1 Yes  99 Prefer not to say |  |
| 3304 | **Liberia ONLY**  Where or who [NAME] got treatment from? Any other place?  SELECT ALL MENTIONED. | 1 Hospital/clinic  2 Drug store  3 Tablet man/black bagger  4 Country doctor  5 CHV  6 Church yard  7 At home  96 Other (please specify) |  |
| 3305 | **Liberia ONLY**  [999 = don’t know, 888 = declined to answer]  When the sickness started, how many days it took before [NAME] get treatment? | [Integer] Days |  |
| 3306 | **Liberia ONLY**  What kind of treatment you gave [NAME]?  SELECT ALL MENTIONED. | 1 Plumpy nut  2 Enriched milk  3 Palm oil  4 Country medicine  96 Other (please specify)  98 Don’t know  99 Prefer not to say |  |
| 3306_spy | **Liberia ONLY**  Please specify | [Text] | [IF 3306=96] |

**END**

**IMPACT INITIATIVE HOUSEHOLD SURVEY**

# MODULE 4: MEN’S QUESTIONNAIRE

**VERSION 3 05/09/19**

X: INTERVIEWER DETAILS

| X1 | Interviewer name | [Drop down list of interviewers] |  |
| --- | --- | --- | --- |
| X2 | Village name | [Drop down list of villages] |  |
| X3 | Segement ID | [ID number of the segment] |  |
| X4 | Name of respondent | [Text] |  |
| X5 | Geocode | [Capture GPS location] |  |

A: INTERVIEW ATTEMPTS

| A1.1 | Date of interview (1^st^) | [Date entry] |  |
| --- | --- | --- | --- |
| A1.2 | Start time (1^st^) | [Time entry] |  |
| A1.3 | Result of interview (1^st^) | 1. Proceeding  2. Postponed  3. Refused | -> B1  -> A2.1  -> |
| A2.1 | Date of interview (2^nd^) | [Date entry] |  |
| A2.2 | Start time (2^nd^) | [Time entry] |  |
| A2.3 | Result of interview (2^nd^) | 1. Proceeding  2. Postponed  3. Refused | -> B1  -> A3.1  -> |
| A3.1 | Date of interview (3^rd^) | [Date entry] |  |
| A3.2 | Start time (3^rd^) | [Time entry] |  |
| A3.3 | Result of interview (3^rd^) | 1. Proceeding  3. Refused | -> B1  -> |

B: PARTICIPANT INFORMATION & CONSENT

[TBC]

3: HEALTH CARE USE, ACUTE CONDITIONS

[Survey form will autofill [NAME] with each household member’s name and cycle through the household roster]

NOTE 2

We would now like to ask you about recent health conditions members of your household may have experienced recently.

| 2101 | Have you suffered from any of the following conditions in the last two weeks (or one month)? [SELECT ALL THAT APPLY] | [TBC]  1 Diarrhoea  2 Fever  3 Difficulty breathing/coughing  4 Serious injury  5 Pain  6 Skin problem (ulcers/sores/rashes etc)  7 Anxiety/depression/difficulty sleeping  8 Nausea/dizziness/light-headed  9 Appetite problems  10 Fatigue  96 Other (please specify) | -> 2102  [If none selected] -> 2201 |
| --- | --- | --- | --- |
| 2101_spy | Please specify | [Text] | [IF 2101=96] |
| 2102 | What was the nature of the injury? | 1 Road traffic accident  2 Fall or other blunt force  3 Poisoning  4 Burn  96 Other (please specify) | [ONLY ASK IF 2101=4]  ->2104  -> 2102_spy |
| 2102_spy | Please specify | [Text] |  |
| 2103 | How serious was the injury? | 2 Permanent disability (such as blindness, deafness, loss of ability to walk)  3 Requiring hospitalisation of 10 days or more  4 Requiring hospitalisation of less than 10 days  5 Requiring medical care and resulting in missed work or school  6 None of the above | [ONLY ASK IF 2101=4] |
| 2104 | Did you seek any care for this condition? | 0 No  1 Yes | -> 2105  -> 2106 |
| 2105 | Why did you not seek care for this condition? | 1 Could not afford the cost of the visit  2 No transport available  3 Could not afford the transport  4 Previous bad treatment  5 Could not take time off work or had other commitments  6 The health care provider’s drugs or equipment were inadequate  7 The health care provider’s skills were inadequate  8 Did not know where to go  9 Tried but were denied health care  10 Was not sick enough  11 Was too sick  12 Did not have permission  13 A lack of privacy at the provider  14 Provider was not open when needed | [IF 2104 = 0]  [Any response] -> 2201 |
| 2106 | Where did you seek care? | **Sierra Leone**  1 Hospital or clinic  2 Drug store  3 Drug peddler  4 Traditional doctor  5 CHW  6 Church yard  7 At home  96 Other (please specify)  **Liberia**  1 Hospital/clinic  2 Drug store  3 Tablet man/black bagger  4 Country doctor  5 CHV  6 Church yard  7 At home  96 Other (please specify) |  |
| 2106_spy | Please specify | [Text] | [IF 1106=96] |
| 2107 | Please name the hospital/clinic | [Dropdown list] | [IF 1106=1 |
| 2107B | Did this visit involve an overnight stay? | 0 No  1 Yes |  |
| 2108 | About how long did it take you to get there? | [Integer] Hours  [Integer] Minutes  [Autofill 0] |  |
| 2109 | How did you get there?  [SELECT ALL THAT APPLY] | 1 Private vehicle  2 Public transportation  3 Taxicab  4 Ambulance or emergency vehicle  5 Bicycle  6 Motorbike  7 Walked  96 Other |  |
| 2110 | About how long did the visit take once you had arrived? [Including waiting times for consultation and treatment] | [Integer] Days  [Integer] Hours  [Integer] Minutes  [Autofill 0] |  |
| 2111 | How much did you pay overall for this visit? [Including transport, consultation fees, drugs, and any tests or services] [OVERALL NET COST LESS ANY MONEY RECEIVED] | [Integer] Leones/LD |  |
| 2112 | Why did you choose this provider? [SELECT ALL THAT APPLY] | 1 Nearness of the facility  2 Service providers are nice/friendly  3 Good services are available  4 Short waiting times  5 Qualified doctors are available  6 Low fees/low treatment cost  7 Good waiting arrangements  8 Confidentiality is maintained  9 Do not know where else to go  10 Medicine is also available  11 Availability of diagnostic service  12 Recommendation from someone  96 Other (please specify) |  |
| 2112_spy | Please specify | [Text] | [IF 2112=96] |
| 2113 | Were there any problems with the visit or service? [SELECT ALL THAT APPLY] | 1 Waited too long  2 Inadequate explanation about the problem or treatment  3 Lack of privacy from having other see or hear the examination or visit  4 Lack of medicines  5 Opening hours are inconvenient  6 Opening days are inconvenient  7 Facility is not clean  8 Poor treatment from staff  9 High cost for services or treatments |  |

4: HEALTH CARE USE, CHRONIC CONDITIONS

NOTE 3

We would now like to ask you about long-term conditions that you might have

| 2201 | Do you have a long-term health conditions expected to last 12 months or more? | 0 No  1 Yes | -> 2301  -> 2202 |
| --- | --- | --- | --- |
| 2202 | What condition do you have?  [SELECT ALL THAT APPLY] | 1 Hypertension  2 Diabetes  3 TB  4 Epilepsy  5 HIV  6 Sickle cell  7 Stroke  96 Other (specify)  98 Don’t know  99 Prefer not to say |  |
| 2202_spy | Please specify | [Text] | [IF 2202=96] |
| 2203 | Have you received any health care for this condition in the last three months? | 0 No  1 Yes | -> 2204  -> 2205 |
| 2204 | Why have you not sought health care for this condition? | 1 Could not afford the cost of the visit  2 No transport available  3 Could not afford the transport  4 Previous bad treatment  5 Could not take time off work or had other commitments  6 The health care provider’s drugs or equipment were inadequate  7 The health care provider’s skills were inadequate  8 Did not know where to go  9 Tried but were denied health care  10 Was not sick enough  11 Was too sick  12 Did not have permission  13 A lack of privacy at the provider  14 Provider was not open when needed | [Any response] -> 2301 |
| 2205 | Where did you receive care for this condition last time? | **Sierra Leone**  1 Hospital or clinic  2 Drug store  3 Drug peddler  4 Traditional doctor  5 CHW  6 Church yard  7 At home  96 Other (please specify)  **Liberia**  1 Hospital/clinic  2 Drug store  3 Tablet man/black bagger  4 Country doctor  5 CHV  6 Church yard  7 At home  96 Other (please specify) |  |
| 2205_spy | Please specify | [Text] | [IF 2205=96] |
| 2206 | Please name the hospital or clinic | [Dropdown list] | [IF 2205=1] |
| 2207 | About how long did it take you to get there? | [Integer] Hours  [Integer] Minutes  [Autofill 0] |  |
| 2208 | How did you get there?  [SELECT ALL THAT APPLY] | 1 Private vehicle  2 Public transportation  3 Taxicab  4 Ambulance or emergency vehicle  5 Bicycle  6 Motorbike  7 Walked  96 Other |  |
| 2209 | About how long did the visit take once you had arrived? [Including waiting times for consultation and treatment] | [Integer] Days  [Integer] Hours  [Integer] Minutes  [Autofill 0] |  |
| 2210 | How much did you pay overall for this visit? [Including transport, consultation fees, drugs, and any tests or services] | [Integer] Leones/LD |  |
| 2211 | Why did you choose this provider? [SELECT ALL THAT APPLY] | 1 Nearness of the facility  2 Service providers are nice/friendly  3 Good services are available  4 Short waiting times  5 Qualified doctors are available  6 Low fees/low treatment cost  7 Good waiting arrangements  8 Confidentiality is maintained  9 Do not know where else to go  10 Medicine is also available  11 Availability of diagnostic service  12 Recommendation from someone  96 Other (please specify) |  |
| 2211_spy | Please specify | [Text] | [IF 2211=96] |
| 2212 | Were there any problems with the visit or service? [SELECT ALL THAT APPLY] | 1 Waited too long  2 Inadequate explanation about the problem or treatment  3 Lack of privacy from having other see or hear the examination or visit  4 Lack of medicines  5 Opening hours are inconvenient  6 Opening days are inconvenient  7 Facility is not clean  8 Poor treatment from staff  9 High cost for services or treatments |  |

1:

OTHER HEALTHCARE USE

| 21XX | How many times in total in the past year have you received health care not including an overnight stay? | [INTEGER] |  |
| --- | --- | --- | --- |
| 21XX | How many times in total in the past year have you been a patient in a hospital or other facility for at least one night? | [INTEGER] |  |
| 22XX | Thinking about the last time you received healthcare, have you already described this visit to me? | 0 No  1 Yes | IF NO USE IN SECTIONS 1 AND 2 |
| 22XX | Thinking about the last time you received healthcare, where did you receive care? | **Sierra Leone**  1 Hospital or clinic  2 Drug store  3 Drug peddler  4 Traditional doctor  5 CHW  6 Church yard  7 At home  96 Other (please specify)  **Liberia**  1 Hospital/clinic  2 Drug store  3 Tablet man/black bagger  4 Country doctor  5 CHV  6 Church yard  7 At home  96 Other (please specify) | IF PREVIOUS QUESTION IS 1 AND EITHER OF FIRST 2 QUESTIONS >0 |
| 22XX | What reason(s) best describe why you needed care? | 1 Diarrhoea  2 Fever  3 Difficulty breathing/coughing  4 Serious injury  5 Pain  6 Skin problem (ulcers/sores/rashes etc)  7 Anxiety/depression/difficulty sleeping  8 Nausea/dizziness/light-headed  9 Appetite problems  10 Fatigue  11 Routine or follow-up visit (i.e. check-up, regular clinic, or treatment for previously diagnosed condition)  96 Other (please specify) |  |
| 22XX_spy | Please specify | [Text] |  |
| 22XX | Please name the hospital or clinic | [Dropdown list] |  |
| 22xx | Did this visit involve an overnight stay? | 0 No  1 Yes | IF NOT AT HOME |
| 22xx | Approximately how long ago was this visit? | [INTEGER] Months |  |
| 22XX | About how long did it take you to get there? | [Integer] Hours  [Integer] Minutes  [Autofill 0] |  |
| 22Xx | How did you get there?  [SELECT ALL THAT APPLY] | 1 Private vehicle  2 Public transportation  3 Taxicab  4 Ambulance or emergency vehicle  5 Bicycle  6 Motorbike  7 Walked  96 Other |  |
| 22XX | About how long did the visit take once you had arrived? [Including waiting times for consultation and treatment] | [Integer] Days  [Integer] Hours  [Integer] Minutes  [Autofill 0] |  |
| 22XX | How much did you pay overall for this visit? [Including transport, consultation fees, drugs, and any tests or services] | [Integer] Leones/LD |  |
| 22XX | Why did you choose this provider? [SELECT ALL THAT APPLY] | 1 Nearness of the facility  2 Service providers are nice/friendly  3 Good services are available  4 Short waiting times  5 Qualified doctors are available  6 Low fees/low treatment cost  7 Good waiting arrangements  8 Confidentiality is maintained  9 Do not know where else to go  10 Medicine is also available  11 Availability of diagnostic service  12 Recommendation from someone  96 Other (please specify) |  |
| 22XX_spy | Please specify | [Text] |  |
| 22XX | Were there any problems with the visit or service? [SELECT ALL THAT APPLY] | 1 Waited too long  2 Inadequate explanation about the problem or treatment  3 Lack of privacy from having other see or hear the examination or visit  4 Lack of medicines  5 Opening hours are inconvenient  6 Opening days are inconvenient  7 Facility is not clean  8 Poor treatment from staff  9 High cost for services or treatments |  |

3: FAMILY PLANNING

NOTE I would like to talk about family planning - the various ways or methods that a couple can use to delay or avoid a pregnancy.

| NOTE | Have you ever heard of the following methods? | NA |  |
| --- | --- | --- | --- |
| 2301A | Female Sterilization. PROBE: Women can have an operation to avoid having any more children. | 0 No  1 Yes |  |
| 2301B | Male Sterilization. PROBE: Men can have an operation to avoid having any more children. | 0 No  1 Yes |  |
| 2301C | IUD. PROBE: Women can have a loop or coil placed inside them by a doctor or a nurse. | 0 No  1 Yes |  |
| 2301D | Injectables. PROBE: Women can have an injection by a health  provider that stops them from becoming pregnant for one or more months. | 0 No  1 Yes |  |
| 2301E | Implants. PROBE: Women can have one or more small rods  placed in their upper arm by a doctor or nurse which can prevent pregnancy for one or more years. | 0 No  1 Yes |  |
| 2301F | Pill. PROBE: Women can take a pill every day to avoid becoming pregnant. | 0 No  1 Yes |  |
| 2301G | Condom. PROBE: Men can put a rubber sheath on their penis before sexual intercourse. | 0 No  1 Yes |  |
| 2301H | Female Condom. PROBE: Women can place a sheath in their vagina before sexual intercourse. | 0 No  1 Yes |  |
| 2301I | Lactational Amenorrhea Method (LAM). | 0 No  1 Yes |  |
| 2301J | Rhythm Method. PROBE: To avoid pregnancy, women do not have  sexual intercourse on the days of the month they think they can get pregnant. | 0 No  1 Yes |  |
| 2301K | Withdrawal. PROBE: Men can be careful and pull out before climax. | 0 No  1 Yes |  |
| 2302 | Are you or your partner currently doing something or using any method to avoid getting pregnant? | 0 No  1 Yes | [NOT IF 2209=1] |
| 2303 | Which method(s) are you using?  [SELECT ALL THAT APPLY] | 1 Female sterilization  2 Male sterilization  3 IUD  4 Injectables  5 Implants  6 Pill  7 Condom  8 Emergency contraception  9 Standard Days Method  10 Lactational Amenorrhea Method  11 Withdrawal | [IF 2302=1] |
| 2304 | Where did you or do you obtain the family planning methods you use? | **Sierra Leone**  1 Hospital or clinic  2 Drug store  3 Drug peddler  4 Traditional doctor/birth attendant  5 CHW  6 Church yard  7 At home  96 Other (please specify)  **Liberia**  1 Hospital/clinic  2 Drug store  3 Tablet man/black bagger  4 Country doctor/traditional birth attendant  5 CHV  6 Church yard  7 At home  96 Other (please specify) | - |
| 2304_spy | Please specify | [Text] | [IF 2304=96] |
| 2305 | In the last few months, have you discussed family planning with a health worker of health professional? | 0 No  1 Yes  98 Don’t know |  |
| 2306A-B | I will now read you some statements about contraception. Please tell me if you agree or disagree with each one. | A Contraception is a woman’s concern and a man should not have to worry about it.  1 Agree  2 Disagree  B Women who use contraception may become promiscuous.  1 Agree  2 Disagree |  |

4: HEALTH AND HEALTH HISTORY

| 2401 | In general would you say your health is: | 1 Excellent  2 Very good  3 Good  4 Fair  5 Poor |  |
| --- | --- | --- | --- |
| 2402 | Compared to one year ago, how would you rate your health in general now? | 1 Much better than a year ago  2 Somewhat better than a year ago  3 About the same  4 Somewhat worse than one year ago  5 Much worse than one year ago |  |
| 2403 | The following question asks how satisfied you feel, on a scale from 0 to 10.  Zero means you feel ‘not at all satisfied’, and 10 means you feel ‘completely satisfied’.  Overall, how satisfied are you with life as a whole these days? | [Integer 0 to 10] |  |
| 2404 | The following question asks how worthwhile you feel the things you do in your life are, on a scale from 0 to 10.  Zero means you feel the things you do in your life are ‘not at all worthwhile’, and 10 means ‘completely worthwhile’.  Overall, to what extent do you feel the things you do in your life are worthwhile? | [Integer 0 to 10] |  |
| NOTE | The following questions ask about how you felt yesterday on a scale from 0 to 10.  Zero means you did not experience the feeling ‘at all’ yesterday, while 10 means you experienced the feeling ‘all of the time’ yesterday. | NA |  |
| 2405 | How about happy? | [Integer 0 to 10] |  |
| 2406 | How about worried? | [Integer 0 to 10] |  |
| 2407 | How about depressed? | [Integer 0 to 10] |  |
| NOTE | HYPERTENSION |  |  |
| 2409 | Have you had your blood pressure checked in the last 12 months by the doctor or any other health care provider? | 0 No  1 Yes  98 Don’t know |  |
| 2410 | Have you ever had your blood pressure measured by the doctor or any other health care provider? | 0 No  1 Yes  98 Don’t know | [IF 2409 = 0] |
| 2411 | Has any health care worker ever told you that you have high blood pressure or hypertension? | 0 No  1 Yes | [IF 2409 = 1 OR 2410 = 1] |
| 2412 | In the past 12 months, Has any health care worker told you that you have high blood pressure or hypertension? | 0 No  1 Yes | [IF 2411=1] |
| 2413 | Has a doctor or other healthcare worker prescribed  medication to control your blood pressure? | 0 No  1 Yes | [IF 2411=1 OR 2412=1] |
| 2414 | Are you taking medication to control your blood  pressure? | 0 No  1 Yes | [IF 2413=1] |
| 2415 | Have you ever seen a traditional healer for high blood pressure or hypertension? | 0 No  1 Yes | [IF 2411 =1] |
| 2416 | Are you currently receiving any of the following for your high blood pressure? | 1 Medication  2 Advice to change diet  3 Advice to lose weight  4 Advice to do more exercise  5 Herbs or traditional remedy  6 Advice to conduct more prayers  96 Other (please specify)  99 Prefer not to say | [IF 2411 =1] |
| NOTE | DIABETES |  |  |
| 2417 | Have you had a finger stick blood sugar test in the last 12 months? | 0 No  1 Yes  98 Don’t Know |  |
| 2418 | Has a health professional ever measured your blood sugar? | 0 No  1 Yes  98 Don’t know | [IF 2417 = 0 OR 99] |
| 2419 | Has a health professional ever told you that you have high blood sugar or diabetes? | 0 No  1 Yes | [IF 2417 = 1 OR 99 OR 2418 = 1 OR 99] |
| 2420 | In the past 12 months, Has a health professional told you that you have high blood sugar or diabetes? | 0 No  1 Yes | [IF 2419=1] |
| 2421 | Have you seen a traditional healer for diabetes or raised blood sugar? | 0 No  1 Yes | [IF 2419 =1] |
| 2422 | Has a doctor or other healthcare worker prescribed  medication to control your high blood sugar or  diabetes? | 0 No  1 Yes | [IF 2419 =1] |
| 2423 | Are you currently taking insulin (injecting yourself or by someone else) for diabetes prescribed by a doctor or health worker? | 0 No  1 Yes | [IF 2419=1] |
| NOTE | MENTAL HEALTH  **LIBERIA ONLY (1424-1434)** |  |  |
| 2424 | Now I’d like to ask you a few questions about mental health. Have you ever sought care for a mental health problem? | 0 No  1 Yes |  |
| 2425 | What was the mental health problem? | 1 Epilepsy  2 Schizophrenia  3 Psychosis  4 Depression  96 Other (please specify)  98 Prefer not to say |  |
| 2425_spy | Please specify | [Text] | [IF 2425=96] |
| 2426 | Were you treated for this problem? | 0 No  1 Yes  98 Prefer not to say |  |
| 2427 | Where or who you get this treatment from?  SELECT ALL MENTIONED | 1 Hospital/clinic  2 Country doctor  3 Friend  4 Family member  5 Community group  6 Church/religious group/leader |  |
| 2428 | What kind of treatment they give you?  SELECT ALL MENTIONED | 1 Medication  2 Counselling  3 Physical exam  4 Surgery  5 Emotional support  6 Plants or herbs  7 Prayer  8 Other (Please specify)  98 Prefer not to say |  |
| 2429 | If you felt that your health might be suffering as a result of stress or strain in your life, would you consider consulting any of these people?  **READ LIST; SELECT ALL MENTIONED** | 1 Friends/family  2 Health professional at hospital/clinic  3 Country doctor/zoe  4 CHV  5 Religious group/leader  99 Prefer not to say |  |
|  | MENTAL HEALTH (CONT.)  **BOTH SITES** |  |  |
| 2430 | Have you ever had any attacks of shaking/jerking of the arms or legs or fallen to the ground and lost consciousness? | 0 No  1 Yes |  |
| 2431 | Has any health care provider ever told you that you have epilepsy? | 0 No  1 Yes |  |
| 2432 | Have you seen a traditional healer for the epilepsy | 0 No  1 Yes |  |
| 2433 | Has a doctor or other healthcare worker prescribed  medication for your epilepsy? | 0 No  1 Yes | [IF 2431=1] |
| 2434 | Where or who did you get that treatment from? | **Sierra Leone**  1 Hospital or clinic  2 Drug store  3 Drug peddler  4 Traditional doctor/birth attendant  5 CHW  6 Church yard  7 At home  96 Other (please specify)  **Liberia**  1 Hospital/clinic  2 Drug store  3 Tablet man/black bagger  4 Country doctor/traditional birth attendant  5 CHV  6 Church yard  7 At home  96 Other (please specify) | [IF 2431 = 1 AND 2433 = 1]  -> 2321_spy |
| 2434_spy | Please specify | [Text] | [if 2434=96] |
| NOTE | TUBERCULOSIS  **LIBERIA ONLY (2435-2443)** |  |  |
| 2435 | Have you ever heard of a sickness called tuberculosis (TB)? | 0 No  1 Yes |  |
| 2436 | What do you know about how this sickness called TB is spread?  [SELECT ALL MENTIONED] | 1 Touching someone with TB  2 Sharing food with someone with TB  3 Coughing  4 Spitting  5 Man woman business  6 Witchcraft  96 Other (please specify)  98 Don’t know  99 Prefer not to say | [IF 2435=1] |
| 2437 | Have you ever had a health professional test you for TB? | 0 No  1 Yes  98 Don’t know  99 Prefer not to say | [IF 2435=1] |
| 2438 | You get the test result? | 0 No  1 Yes  99 Prefer not to say | [IF 2437=1] |
| 2439 | What was the test result? | 0 Negative  1 Positive  99 Prefer not to say | [IF 2438=1] |
| 2440 | Where and who you get treatment for TB from? Any other place?  SELECT ALL MENTIONED | 1 Hospital/clinic  2 Drug store  3 Tablet man/black bagger  4 Country doctor/traditional birth attendant  5 CHV  6 Church yard  7 At home  96 Other (please specify) | [IF 2439=1] |
| 2440_spy | Please specify | [Text] | [IF 2440=96] |
| 2441 | Who did you go for treatment or advice FIRST? | 1 Hospital/clinic  2 Drug store  3 Tablet man/black bagger  4 Country doctor/traditional birth attendant  5 CHV  6 Church yard  7 At home  96 Other (please specify) | [IF ANY OPTION 2440] |
| 2441_spy | Please specify | [Text] | [IF 2441=96] |
| 2442 | What kind of treatment you take for TB? | 1 Tablets from clinician at health facility  2 Country medicine  3 Prayer  96 Other (please specify)  98 Don’t know  99 Prefer not to say | [IF 2439=1] |
| 2443 | Did you take it until the clinician tell you ‘stop’? | 0 No  1 Yes  98 Don’t know  99 Prefer not to say |  |
| NOTE | HIV  **LIBERIA ONLY (2444-END)** |  |  |
| 2444 | Now I want to ask you some questions about HIV. Have you ever heard of a illness called HIV? | 0 No  1 Yes  99 Prefer not to say |  |
| 2445 | Can people get HIV from mosquito bites? | 0 No  1 Yes  98 Don’t know |  |
| 2446 | Can people get HIV by sharing food with a person who has HIV? | 0 No  1 Yes  98 Don’t know |  |
| 2447 | Can people get HIV from witchcraft or something like that? | 0 No  1 Yes  98 Don’t know |  |
| 2448 | Is it true that a healthy-looking person can have HIV? | 0 No  1 Yes  98 Don’t know |  |
| 2449 | If a member of your family/partner got infected with HIV, would you want it to remain a secret or not? | 0 No  1 Yes  98 Don’t know  99 Prefer not to say |  |
| 2450 | If a member of your family/partner became sick from HIV, would you be willing to care for her or him in your own household? | 0 No  1 Yes  98 Don’t know  99 Prefer not to say |  |
| 2451 | Would you buy fresh vegetables from someone at the market if you knew that this person got HIV? | 0 No  1 Yes  98 Don’t know  99 Prefer not to say |  |
| 2452 | In your opinion, if a teacher has HIV but is not sick, should he/she be allowed to continue teaching in the school? | 0 No  1 Yes  98 Don’t know  99 Prefer not to say |  |
| 2453 | Can HIV be transmitted from a mother to her baby during pregnancy? | 0 No  1 Yes  98 Don’t know |  |
| 2454 | Can HIV be transmitted from a mother to her baby during pregnancy? | 0 No  1 Yes  98 Don’t know |  |
| 2455 | Can HIV be transmitted from a mother to her baby during pregnancy? | 0 No  1 Yes  98 Don’t know |  |
| 2456 | Are there any special drugs that a doctor or a nurse can give to a women infected with HIV to reduce the risk of transmission to the baby? | 0 No  1 Yes  98 Don’t know  99 Prefer not to say |  |
| 2457 | I don’t want to know the results, but have you ever been tested to see if you are HIV positive? | 0 No  1 Yes  98 Don’t know  99 Prefer not to say |  |
| 2458 | How many months ago was your most recent HIV test?  ENTER 25 IF GREATER THAN 2 YEARS OR 99 IF THE PATIENT DECLINED TO ANSWER | [Integer]  [Max of 25] | [IF 2457=1] |
| 2459 | I don’t want to know the results, but did you get the results of the test? | 0 No  1 Yes  99 Prefer not to say |  |
| 2460 | Where you get this test done? | 1 Hospital/clinic  2 Drug store  3 Tablet man/black bagger  4 Country doctor/traditional birth attendant  5 CHV  6 Church yard  7 At home  96 Other (please specify) |  |
| 2460_spy | Please specify |  |  |

**END**

**IMPACT INITATIVE HOUSEHOLD SURVEY**

# MODULE 4: WOMEN’S QUESTIONNAIRE

**VERSION 3 05/09/19**

X: INTERVIEWER DETAILS

| X1 | Interviewer name | [Drop down list of interviewers] |  |
| --- | --- | --- | --- |
| X2 | Village name | [Drop down list of villages] |  |
| X3 | Segment ID | [ID number of the segment] |  |
| X4 | Name of respondent | [Text] |  |
| X5 | Geocode | [Capture GPS location] |  |

A: INTERVIEW ATTEMPTS

| A1.1 | Date of interview (1^st^) | [Date entry] |  |
| --- | --- | --- | --- |
| A1.2 | Start time (1^st^) | [Time entry] |  |
| A1.3 | Result of interview (1^st^) | 1. Proceeding  2. Postponed  3. Refused | -> B1  -> A2.1  -> |
| A2.1 | Date of interview (2^nd^) | [Date entry] |  |
| A2.2 | Start time (2^nd^) | [Time entry] |  |
| A2.3 | Result of interview (2^nd^) | 1. Proceeding  2. Postponed  3. Refused | -> B1  -> A3.1  -> |
| A3.1 | Date of interview (3^rd^) | [Date entry] |  |
| A3.2 | Start time (3^rd^) | [Time entry] |  |
| A3.3 | Result of interview (3^rd^) | 1. Proceeding  3. Refused | -> B1  -> |

B: PARTICIPANT INFORMATION & CONSENT

[TBC]

3: HEALTH CARE USE, ACUTE CONDITIONS

[Survey form will autofill [NAME] with each household member’s name and cycle through the household roster]

NOTE 2

We would now like to ask you about recent health conditions members of your household may have experienced recently.

| 1101 | Have you suffered from any of the following conditions in the last two weeks? [SELECT ALL THAT APPLY] | 1 Diarrhoea  2 Fever  3 Difficulty breathing/coughing  4 Serious injury  5 Pain  6 Skin problem (ulcers/sores/rashes etc)  7 Anxiety/depression/difficulty sleeping  8 Nausea/dizziness/light-headed  9 Appetite problems  10 Fatigue  96 Other (please specify) | -> 1102  [If none selected] -> 1201 |
| --- | --- | --- | --- |
| 1101_spy | Please specify | [Text] | [IF 1101=96] |
| 1102 | What was the nature of the injury? | 1 Road traffic accident  2 Fall or other blunt force  3 Poisoning  4 Burn  96 Other (please specify) | [ONLY ASK IF 1101=4]  ->1104  -> 1102_spy |
| 1102_spy | Please specify | [Text] |  |
| 1103 | How serious was the injury? | 1 Fatal  2 Permanent disability (such as blindness, deafness, loss of ability to walk)  3 Requiring hospitalisation of 10 days or more  4 Requiring hospitalisation of less than 10 days  5 Requiring medical care and resulting in missed work or school  6 None of the above | [ONLY ASK IF 1101=4] |
| 1104 | Did you seek any care for this condition? | 0 No  1 Yes | -> 1105  -> 1106 |
| 1105 | Why did you not seek care for this condition? | 1 Could not afford the cost of the visit  2 No transport available  3 Could not afford the transport  4 Previous bad treatment  5 Could not take time off work or had other commitments  6 The health care provider’s drugs or equipment were inadequate  7 The health care provider’s skills were inadequate  8 Did not know where to go  9 Tried but were denied health care  10 Was not sick enough  11 Was too sick  12 Did not have permission | [IF 1104 = 0]  [Any response] -> 1201 |
| 1106 | Where did you seek care? | **Sierra Leone**  1 Hospital or clinic  2 Drug store  3 Drug peddler  4 Traditional doctor  5 CHW  6 Church yard  7 At home  96 Other (please specify)  **Liberia**  1 Hospital/clinic  2 Drug store  3 Tablet man/black bagger  4 Country doctor  5 CHV  6 Church yard  7 At home  96 Other (please specify) |  |
| 1106_spy | Please specify | [Text] | [IF 1106=96] |
| 1107 | Please name the hospital/clinic | [Dropdown list] | [IF 1106=1 |
| 1108 | About how long did it take you to get there? | [Integer] Hours  [Integer] Minutes  [Autofill 0] |  |
| 1109 | How did you get there?  [SELECT ALL THAT APPLY] | 1 Private vehicle  2 Public transportation  3 Taxicab  4 Ambulance or emergency vehicle  5 Bicycle  6 Motorbike  7 Walked  96 Other |  |
| 1110 | About how long did the visit take once you had arrived? [Including waiting times for consultation and treatment] | [Integer] Days  [Integer] Hours  [Integer] Minutes  [Autofill 0] |  |
| 1111 | How much did you pay overall for this visit? [Including transport, consultation fees, drugs, and any tests or services] [OVERALL NET COST LESS ANY MONEY RECEIVED] | [Integer] Leones/LD |  |
| 1112 | Why did you choose this provider? [SELECT ALL THAT APPLY] | 1 Nearness of the facility  2 Service providers are nice/friendly  3 Good services are available  4 Short waiting times  5 Qualified doctors are available  6 Low fees/low treatment cost  7 Good waiting arrangements  8 Confidentiality is maintained  9 Do not know where else to go  10 Medicine is also available  11 Availability of diagnostic service  12 Recommendation from someone  96 Other (please specify) |  |
| 1112_spy | Please specify | [Text] | [IF 1112=96] |
| 1113 | Were there any problems with the visit or service? [SELECT ALL THAT APPLY] | 1 Waited too long  2 Inadequate explanation about the problem or treatment  3 Lack of privacy from having other see or hear the examination or visit  4 Lack of medicines  5 Opening hours are inconvenient  6 Opening days are inconvenient  7 Facility is not clean  8 Poor treatment from staff  9 High cost for services or treatments |  |

4: HEALTH CARE USE, CHRONIC CONDITIONS

NOTE 3

We would now like to ask you about long-term conditions that you might have

| 1201 | Do you have a long-term health conditions? | 0 No  1 Yes | -> 1301  -> 1202 |
| --- | --- | --- | --- |
| 1202 | What condition do you have?  [SELECT ALL THAT APPLY] | 1 Hypertension  2 Diabetes  3 TB  4 Epilepsy  5 HIV  6 Sickle cell  7 Stroke  96 Other (specify)  98 Don’t know  99 Prefer not to say |  |
| 1202_spy | Please specify | [Text] | [IF 1202=96] |
| 1203 | Have you received any health care for this condition in the last three months? | 0 No  1 Yes | -> 1204  -> 1205 |
| 1204 | Why have you not sought health care for this condition? | 1 Could not afford the cost of the visit  2 No transport available  3 Could not afford the transport  4 Previous bad treatment  5 Could not take time off work or had other commitments  6 The health care provider’s drugs or equipment were inadequate  7 The health care provider’s skills were inadequate  8 Did not know where to go  9 Tried but were denied health care  10 Was not sick enough  11 Was too sick  12 Did not have permission | [Any response] -> 1301 |
| 1205 | Where did you receive care for this condition last time? | **Sierra Leone**  1 Hospital or clinic  2 Drug store  3 Drug peddler  4 Traditional doctor  5 CHW  6 Church yard  7 At home  96 Other (please specify)  **Liberia**  1 Hospital/clinic  2 Drug store  3 Tablet man/black bagger  4 Country doctor  5 CHV  6 Church yard  7 At home  96 Other (please specify) |  |
| 1205_spy | Please specify | [Text] | [IF 1205=96] |
| 1206 | Please name the hospital or clinic | [Dropdown list] | [IF 1205=1] |
| 1207 | About how long did it take you to get there? | [Integer] Hours  [Integer] Minutes  [Autofill 0] |  |
| 1208 | How did you get there?  [SELECT ALL THAT APPLY] | 1 Private vehicle  2 Public transportation  3 Taxicab  4 Ambulance or emergency vehicle  5 Bicycle  6 Motorbike  7 Walked  96 Other |  |
| 1209 | About how long did the visit take once you had arrived? [Including waiting times for consultation and treatment] | [Integer] Days  [Integer] Hours  [Integer] Minutes  [Autofill 0] |  |
| 1210 | How much did you pay overall for this visit? [Including transport, consultation fees, drugs, and any tests or services] | [Integer] Leones/LD |  |
| 1211 | Why did you choose this provider? [SELECT ALL THAT APPLY] | 1 Nearness of the facility  2 Service providers are nice/friendly  3 Good services are available  4 Short waiting times  5 Qualified doctors are available  6 Low fees/low treatment cost  7 Good waiting arrangements  8 Confidentiality is maintained  9 Do not know where else to go  10 Medicine is also available  11 Availability of diagnostic service  12 Recommendation from someone  96 Other (please specify) |  |
| 1211_spy | Please specify | [Text] | [IF 1211=96] |
| 1212 | Were there any problems with the visit or service? [SELECT ALL THAT APPLY] | 1 Waited too long  2 Inadequate explanation about the problem or treatment  3 Lack of privacy from having other see or hear the examination or visit  4 Lack of medicines  5 Opening hours are inconvenient  6 Opening days are inconvenient  7 Facility is not clean  8 Poor treatment from staff  9 High cost for services or treatments |  |

1:

PREGNANCY & MATERNAL CONDITIONS

NOTE 1 We would like to ask you about any pregnancies and births you have had in the last year.

| 1301 | Have given birth in the last year? | 0 No  1 Yes |  |
| --- | --- | --- | --- |
| 1302 | What was the outcome of the birth? | 1 Live birth, baby currently living  2 Live birth, baby later died  3 Stillbirth  99 Prefer not to say | [IF 1101 = 1] |
| 1303 | **Sierra Leone**  While pregnant, did you see anyone for antenatal care?  **Liberia**  Did you see anyone for big belly checkups when you had belly with that child (ANC visits)? | 0 No  1 Yes  99 Prefer not to say |  |
| 1304 | Where did you see someone? | **Sierra Leone**  1 Hospital or clinic  2 Drug store  3 Drug peddler  4 Traditional doctor/birth attendant  5 CHW  6 Church yard  7 At home  96 Other (please specify)  **Liberia**  1 Hospital/clinic  2 Drug store  3 Tablet man/black bagger  4 Country doctor/traditional birth attendant  5 CHV  6 Church yard  7 At home  96 Other (please specify) | [IF 1104=1] |
| 1304_spy | Please specify | [Text] |  |
| 1305 | Please name the facility | [Drop down list of facilities] | [IF 1104 = 1] |
| 1306 | Who did you see? | **Sierra Leone**  1 Doctor  2 Nurse  3 Midwife  4 CHO  5 Traditional birth attendant  6 Traditional doctor  7 CHW  96 Other  **Liberia**  1 Doctor  2 Nurse  3 Certified midwife  4 Physician assistant (PA)  5 Trained traditional midwife  6 Country doctor  7 CHV  96 Other |  |
| 1307 | How many months pregnant were you when you first received antenatal care? | [Integer] Months (max 9) | [IF 1103=1] |
| 1308 | How many times did you receive antenatal care? | [Integer] (max 8) | [IF 1103=1] |
| 1309 | Where did you give birth? | 1 Hospital/clinic  2 At home  3 On the way to the hospital/clinic  4 At traditional birth attendant  96 Other (please specify) | [IF 1101 = 1] |
| 1310 | Please name the facility | [Drop down list of facilities] | [If 1109 = 1] |
| 1309_spy | Please specify | [Text] | [IF 1109=96] |
| 1311 | What were the reasons why the child was not born in a hospital or health facility?  [SELECT ALL THAT APPLY] | 1 Could not afford the cost of the visit  2 No transport available  3 Could not afford the transport  4 Previous bad treatment  5 Could not take time off work or had other commitments  6 The health care provider’s drugs or equipment were inadequate  7 The health care provider’s skills were inadequate  8 Did not know where to go  9 Tried but were denied health care  10 Was not sick enough  11 Was too sick  12 Did not have permission |  |
| 1312 | **Liberia ONLY**  After you born the child, did any person check on you quick-quick to see how you were coming on during that time? | 0 No  1 Yes  99 Prefer not to say |  |
| 1313 | **Liberia ONLY**  How long it took after you born the baby before they first check you? | [Integer]  Select unit  1 Hours  2 Days  3 Weeks |  |
| 1314 | **Liberia ONLY**  Where they check on you? | 1 Hospital/clinic  2 Drug store  3 Tablet man/black bagger  4 Country doctor/traditional birth attendant  5 CHV  6 Church yard  7 At home  96 Other (please specify) |  |
| 1314_spy | Please specify | [Text] | [IF 1114 = 96] |
| 1315 | **Liberia ONLY**  Who check on the baby condition at that time? | 1 Doctor  2 Nurse  3 Certified midwife  4 Physician assistant (PA)  5 Trained traditional midwife  6 Country doctor  7 CHV  96 Other |  |
| 1316 | **Liberia ONLY**  If you born the child at the facility, or someone checked on you after you born the child, how respectfully did they treat you? | 1 Respectfully  2 Somewhat respectfully  3 Somewhat disrespectfully  4 Disrespectfully |  |
| 1317 | Are you currently pregnant? | 0 No  1 Yes  99 Prefer not to say |  |
| 1318 | How many months pregnant are you? | [Integer – max of 9] | [IF 1317=2] |

4: FAMILY PLANNING

NOTE Now I would like to talk about family planning - the various ways or methods that a couple can use to delay or avoid a pregnancy.

| NOTE | Have you ever heard of the following methods? | NA |  |
| --- | --- | --- | --- |
| 1401A | Female Sterilization. PROBE: Women can have an operation to avoid having any more children. | 0 No  1 Yes |  |
| 1401B | Male Sterilization. PROBE: Men can have an operation to avoid having any more children. | 0 No  1 Yes |  |
| 1401C | IUD. PROBE: Women can have a loop or coil placed inside them by a doctor or a nurse. | 0 No  1 Yes |  |
| 1401D | Injectables. PROBE: Women can have an injection by a health  provider that stops them from becoming pregnant for one or more months. | 0 No  1 Yes |  |
| 1401E | Implants. PROBE: Women can have one or more small rods  placed in their upper arm by a doctor or nurse which can prevent pregnancy for one or more years. | 0 No  1 Yes |  |
| 1401F | Pill. PROBE: Women can take a pill every day to avoid becoming pregnant. | 0 No  1 Yes |  |
| 1401G | Condom. PROBE: Men can put a rubber sheath on their penis before sexual intercourse. | 0 No  1 Yes |  |
| 1401H | Female Condom. PROBE: Women can place a sheath in their vagina before sexual intercourse. | 0 No  1 Yes |  |
| 1401I | Lactational Amenorrhea Method (LAM). | 0 No  1 Yes |  |
| 1401J | Rhythm Method. PROBE: To avoid pregnancy, women do not have  sexual intercourse on the days of the month they think they can get pregnant. | 0 No  1 Yes |  |
| 1401K | Withdrawal. PROBE: Men can be careful and pull out before climax. | 0 No  1 Yes |  |
| 1402 | Are you or your partner currently doing something or using any method to avoid getting pregnant? | 0 No  1 Yes | [NOT IF 1109=1] |
| 1403 | Which method(s) are you using?  [SELECT ALL THAT APPLY] | 1 Female sterilization  2 Male sterilization  3 IUD  4 Injectables  5 Implants  6 Pill7 Condom  8 Emergency contraception  9 Standard Days Method  10 Lactational Amenorrhea Method  11 Withdrawal | [IF 1202=1] |
| 1404 | Where did you obtain the family planning methods you use? | 1 Hospital or clinic  2 Drug store  3 Drug peddler  4 Traditional doctor  5 CHW  96 Other (please specify) | -> 1204_spy |
| 1404_spy | Please specify | [Text] |  |
| 1405 | The last time you visited a health facility or clinic, did a health worker speak to you about family planning methods? | 0 No  1 Yes  2 I haven’t been to a facility  99 Don’t know |  |
| 1406 | Would you say that using family planning is mainly your decision, your partner’s/husband’s decision, or do you both decide together? | 1 My decision  2 Husband’s decision  3 Joint decision |  |

4: HEALTH AND HEALTH HISTORY

| 1401 | In general would you say your health is: | 1 Excellent  2 Very good  3 Good  4 Fair  5 Poor |  |
| --- | --- | --- | --- |
| 1402 | Compared to one year ago, how would you rate your health in general now? | 1 Much better than a year ago  2 Somewhat better than a year ago  3 About the same  4 Somewhat worse than one year ago  5 Much worse than one year ago |  |
| 1403 | The following question asks how satisfied you feel, on a scale from 0 to 10.  Zero means you feel ‘not at all satisfied’, and 10 means you feel ‘completely satisfied’.  Overall, how satisfied are you with life as a whole these days? | [Integer 0 to 10] |  |
| 1404 | The following question asks how worthwhile you feel the things you do in your life are, on a scale from 0 to 10.  Zero means you feel the things you do in your life are ‘not at all worthwhile’, and 10 means ‘completely worthwhile’.  Overall, to what extent do you feel the things you do in your life are worthwhile? | [Integer 0 to 10] |  |
| NOTE | The following questions ask about how you felt yesterday on a scale from 0 to 10.  Zero means you did not experience the feeling ‘at all’ yesterday, while 10 means you experienced the feeling ‘all of the time’ yesterday. | NA |  |
| 1405 | How about happy? | [Integer 0 to 10] |  |
| 1406 | How about worried? | [Integer 0 to 10] |  |
| 1407 | How about depressed? | [Integer 0 to 10] |  |
| 1408A-D | Many different factors can prevent women from getting medical advice or treatment for themselves. When you are sick and want to get medical advice or treatment, are the following every a problem for you: | A Getting permission to go to the doctor  0 No  1 Yes  B Getting money needed for advice and treatment  etc..  C The distance to the health facility  D Not wanting to go alone |  |
| NOTE | HYPERTENSION |  |  |
| 1409 | Have you had your blood pressure checked in the last 12 months by the doctor or any other health care provider? | 0 No  1 Yes  98 Don’t know |  |
| 1410 | Have you ever had your blood pressure measured by the doctor or any other health care provider? | 0 No  1 Yes  98 Don’t know | [IF 1409 = 0] |
| 1411 | Has any health care worker ever told you that you have high blood pressure or hypertension? | 0 No  1 Yes | [IF 1409 = 1 OR 1410 = 1] |
| 1412 | In the past 12 months, Has any health care worker told you that you have high blood pressure or hypertension? | 0 No  1 Yes | [IF 1411=1] |
| 1413 | Has a doctor or other healthcare worker prescribed  medication to control your blood pressure? | 0 No  1 Yes | [IF 1411=1 OR 1412=1] |
| 1414 | Are you taking medication to control your blood  pressure? | 0 No  1 Yes | [IF 1413=1] |
| 1415 | Have you ever seen a traditional healer for high blood pressure or hypertension? | 0 No  1 Yes | [IF 1411 =1] |
| 1416 | Are you currently receiving any of the following for your high blood pressure? | 1 Medication  2 Advice to change diet  3 Advice to lose weight  4 Advice to do more exercise  5 Herbs or traditional remedy  6 Advice to conduct more prayers  96 Other (please specify)  99 Prefer not to say | [IF 1411 =1] |
| NOTE | DIABETES |  |  |
| 1417 | Have you had a finger stick blood sugar test in the last 12 months? | 0 No  1 Yes  98 Don’t Know |  |
| 1418 | Has a health professional ever measured your blood sugar? | 0 No  1 Yes  98 Don’t know | [IF 1417 = 0 OR 99] |
| 1419 | Has a health professional ever told you that you have high blood sugar or diabetes? | 0 No  1 Yes | [IF 1417 = 1 OR 99 OR 1418 = 1 OR 99] |
| 1420 | In the past 12 months, Has a health professional told you that you have high blood sugar or diabetes? | 0 No  1 Yes | [IF 1419=1] |
| 1421 | Have you seen a traditional healer for diabetes or raised blood sugar? | 0 No  1 Yes | [IF 1419 =1] |
| 1422 | Has a doctor or other healthcare worker prescribed  medication to control your high blood sugar or  diabetes? | 0 No  1 Yes | [IF 1419 =1] |
| 1423 | Are you currently taking insulin (injecting yourself or by someone else) for diabetes prescribed by a doctor or health worker? | 0 No  1 Yes | [IF 1419=1] |
| NOTE | MENTAL HEALTH  **LIBERIA ONLY (1424-1434)** |  |  |
| 1424 | Now I’d like to ask you a few questions about mental health. Have you ever sought care for a mental health problem? | 0 No  1 Yes |  |
| 1425 | What was the mental health problem? | 1 Epilepsy  2 Schizophrenia  3 Psychosis  4 Depression  96 Other (please specify)  98 Prefer not to say |  |
| 1425_spy | Please specify | [Text] | [IF 1425=96] |
| 1426 | Were you treated for this problem? | 0 No  1 Yes  98 Prefer not to say |  |
| 1427 | Where or who you get this treatment from?  SELECT ALL MENTIONED | 1 Hospital/clinic  2 Country doctor  3 Friend  4 Family member  5 Community group  6 Church/religious group/leader |  |
| 1428 | What kind of treatment they give you?  SELECT ALL MENTIONED | 1 Medication  2 Counselling  3 Physical exam  4 Surgery  5 Emotional support  6 Plants or herbs  7 Prayer  8 Other (Please specify)  98 Prefer not to say |  |
| 1429 | If you felt that your health might be suffering as a result of stress or strain in your life, would you consider consulting any of these people?  **READ LIST; SELECT ALL MENTIONED** | 1 Friends/family  2 Health professional at hospital/clinic  3 Country doctor/zoe  4 CHV  5 Religious group/leader  99 Prefer not to say |  |
|  | MENTAL HEALTH (CONT.)  **BOTH SITES** |  |  |
| 1430 | Have you ever had any attacks of shaking/jerking of the arms or legs or fallen to the ground and lost consciousness? | 0 No  1 Yes |  |
| 1431 | Has any health care provider ever told you that you have epilepsy? | 0 No  1 Yes |  |
| 1432 | Have you seen a traditional healer for the epilepsy | 0 No  1 Yes |  |
| 1433 | Has a doctor or other healthcare worker prescribed  medication for your epilepsy? | 0 No  1 Yes | [IF 1431=1] |
| 1434 | Where or who did you get that treatment from? | **Sierra Leone**  1 Hospital or clinic  2 Drug store  3 Drug peddler  4 Traditional doctor/birth attendant  5 CHW  6 Church yard  7 At home  96 Other (please specify)  **Liberia**  1 Hospital/clinic  2 Drug store  3 Tablet man/black bagger  4 Country doctor/traditional birth attendant  5 CHV  6 Church yard  7 At home  96 Other (please specify) | [IF 1431 = 1 AND 1433 = 1]  -> 1321_spy |
| 1434_spy | Please specify | [Text] | [if 1434=96] |
| NOTE | TUBERCULOSIS  **LIBERIA ONLY (1435-1443)** |  |  |
| 1435 | Have you ever heard of a sickness called tuberculosis (TB)? | 0 No  1 Yes |  |
| 1436 | What do you know about how this sickness called TB is spread?  [SELECT ALL MENTIONED] | 1 Touching someone with TB  2 Sharing food with someone with TB  3 Coughing  4 Spitting  5 Man woman business  6 Witchcraft  96 Other (please specify)  98 Don’t know  99 Prefer not to say | [IF 1435=1] |
| 1437 | Have you ever had a health professional test you for TB? | 0 No  1 Yes  98 Don’t know  99 Prefer not to say | [IF 1435=1] |
| 1438 | You get the test result? | 0 No  1 Yes  99 Prefer not to say | [IF 1437=1] |
| 1439 | What was the test result? | 0 Negative  1 Positive  99 Prefer not to say | [IF 1438=1] |
| 1440 | Where and who you get treatment for TB from? Any other place?  SELECT ALL MENTIONED | 1 Hospital/clinic  2 Drug store  3 Tablet man/black bagger  4 Country doctor/traditional birth attendant  5 CHV  6 Church yard  7 At home  96 Other (please specify) | [IF 1439=1] |
| 1440_spy | Please specify | [Text] | [IF 1440=96] |
| 1441 | Who did you go for treatment or advice FIRST? | 1 Hospital/clinic  2 Drug store  3 Tablet man/black bagger  4 Country doctor/traditional birth attendant  5 CHV  6 Church yard  7 At home  96 Other (please specify) | [IF ANY OPTION 1440] |
| 1441_spy | Please specify | [Text] | [IF 1441=96] |
| 1442 | What kind of treatment you take for TB? | 1 Tablets from clinician at health facility  2 Country medicine  3 Prayer  96 Other (please specify)  98 Don’t know  99 Prefer not to say | [IF 1439=1] |
| 1443 | Did you take it until the clinician tell you ‘stop’? | 0 No  1 Yes  98 Don’t know  99 Prefer not to say |  |
| NOTE | HIV  **LIBERIA ONLY (1444-** |  |  |
| 1444 | Now I want to ask you some questions about HIV. Have you ever heard of a illness called HIV? | 0 No  1 Yes  99 Prefer not to say |  |
| 1445 | Can people get HIV from mosquito bites? | 0 No  1 Yes  98 Don’t know |  |
| 1446 | Can people get HIV by sharing food with a person who has HIV? | 0 No  1 Yes  98 Don’t know |  |
| 1447 | Can people get HIV from witchcraft or something like that? | 0 No  1 Yes  98 Don’t know |  |
| 1448 | Is it true that a healthy-looking person can have HIV? | 0 No  1 Yes  98 Don’t know |  |
| 1449 | If a member of your family/partner got infected with HIV, would you want it to remain a secret or not? | 0 No  1 Yes  98 Don’t know  99 Prefer not to say |  |
| 1450 | If a member of your family/partner became sick from HIV, would you be willing to care for her or him in your own household? | 0 No  1 Yes  98 Don’t know  99 Prefer not to say |  |
| 1451 | Would you buy fresh vegetables from someone at the market if you knew that this person got HIV? | 0 No  1 Yes  98 Don’t know  99 Prefer not to say |  |
| 1452 | In your opinion, if a teacher has HIV but is not sick, should he/she be allowed to continue teaching in the school? | 0 No  1 Yes  98 Don’t know  99 Prefer not to say |  |
| 1453 | Can HIV be transmitted from a mother to her baby during pregnancy? | 0 No  1 Yes  98 Don’t know |  |
| 1454 | Can HIV be transmitted from a mother to her baby during pregnancy? | 0 No  1 Yes  98 Don’t know |  |
| 1455 | Can HIV be transmitted from a mother to her baby during pregnancy? | 0 No  1 Yes  98 Don’t know |  |
| 1456 | Are there any special drugs that a doctor or a nurse can give to a women infected with HIV to reduce the risk of transmission to the baby? | 0 No  1 Yes  98 Don’t know  99 Prefer not to say |  |
| 1457 | I don’t want to know the results, but have you ever been tested to see if you are HIV positive? | 0 No  1 Yes  98 Don’t know  99 Prefer not to say |  |
| 1458 | How many months ago was your most recent HIV test?  ENTER 25 IF GREATER THAN 2 YEARS OR 99 IF THE PATIENT DECLINED TO ANSWER | [Integer]  [Max of 25] | [IF 1457=1] |
| 1459 | I don’t want to know the results, but did you get the results of the test? | 0 No  1 Yes  99 Prefer not to say |  |
| 1460 | Where you get this test done? | 1 Hospital/clinic  2 Drug store  3 Tablet man/black bagger  4 Country doctor/traditional birth attendant  5 CHV  6 Church yard  7 At home  96 Other (please specify) |  |
| 1460_spy | Please specify |  |  |

**END**
